# Endocytic Markers Associated with the Internalization and Processing of *Aspergillus fumigatus* Conidia by BEAS-2B Cells

**DOI:** 10.1128/mSphere.00663-18

**Published:** 2019-02-06

**Authors:** Helen R. Clark, Allison B. Powell, Kelsey A. Simmons, Tariq Ayubi, Shiv D. Kale

**Affiliations:** a Biocomplexity Institute of Virginia Tech, Blacksburg, Virginia, USA; Carnegie Mellon University

**Keywords:** *Aspergillus fumigatus*, invasive pulmonary aspergillosis, airway epithelial cells, conidia, endocytosis

## Abstract

Aspergillus fumigatus is a ubiquitous mold that produces small airborne conidia capable of traversing deep into the respiratory system. Recognition, processing, and clearance of A. fumigatus conidia by bronchial airway epithelial cells are thought to be relevant to host defense and immune signaling. Using z-stack confocal microscopy, we observed that only 10 to 20% of adherent conidia from the AF293 clinical isolate are internalized by BEAS-2B cells 6 h postchallenge and not prior. Similar percentages of internalization were observed for the CEA10 clinical isolate. A large subset of both AF293 and CEA10 conidia are rendered metabolically inactive without internalization at 3 h postchallenge by BEAS-2B cells. A significantly larger percentage of CEA10 conidia are metabolically active at 9 and 12 h postchallenge in comparison to the AF293 isolate, demonstrating heterogeneity among clinical isolates. We identified 7 host markers (caveolin, flotillin-2, RAB5C, RAB8B, RAB7A, 2xFYVE, and FAPP1) that consistently localized around internalized conidia 9 h postchallenge. Transient gene silencing of *RAB5C*, *PIK3C3*, and *flotillin-2* resulted in a larger population of metabolically active conidia. Our findings emphasize the abundance of both host phosphatidylinositol 3-phosphate (PI3P) and PI4P around internalized conidia, as well as the importance of class III PI3P kinase for conidial processing. Therapeutic development focused on RAB5C-, PIK3C3-, and flotillin-2-mediated pathways may provide novel opportunities to modulate conidial processing and internalization. Determination of how contacted, external conidia are processed by airway epithelial cells may also provide a novel avenue to generate host-targeted therapeutics.

**IMPORTANCE** Conidia from the fungus Aspergillus fumigatus are notorious for their ability to stay airborne. This characteristic is believed to allow conidia to penetrate into the cleanest environments. Several hundred conidia are thought to be inhaled each day by a given individual and then expelled by mucociliary clearance. Given that airway epithelial cells make up a significant portion of the pulmonary-air interface, we set out to determine the percentage of conidia that are actually internalized after initial contact with airway epithelial cells. We determined this through an *in vitro* assay using an immortalized bronchial airway epithelial cell line known as BEAS-2B. Our results suggest a small fraction of conidia are internalized by BEAS-2B cells, while the majority stay adherent to the surface of cells or are washed away during sample processing. Internalization of conidia was observed at 6 h postchallenge and not prior. Our data also indicate conidia are rendered metabolically inactive within 3 h of challenge, suggesting BEAS-2B cells process a large number of conidia without internalization in this early time frame. We have also identified several host endocytosis markers that localize around internalized conidia as well as contribute to the processing of conidia. Understanding how these host endocytosis markers affect the processing of internal and/or external conidia may provide a novel avenue for therapeutic development.

## Abstract (precis)

Conidia from the fungus Aspergillus fumigatus are notorious for their ability to stay airborne. This characteristic is believed to allow conidia to penetrate into the cleanest environments. Several hundred conidia are thought to be inhaled each day by a given individual and then expelled by mucociliary clearance. Given that airway epithelial cells make up a significant portion of the pulmonary-air interface, we set out to determine the percentage of conidia that are actually internalized after initial contact with airway epithelial cells. We determined this through an *in vitro* assay using an immortalized bronchial airway epithelial cell line known as BEAS-2B. Our results suggest a small fraction of conidia are internalized by BEAS-2B cells, while the majority stay adherent to the surface of cells or are washed away during sample processing. Internalization of conidia was observed at 6 h postchallenge and not prior. Our data also indicate conidia are rendered metabolically inactive within 3 h of challenge, suggesting BEAS-2B cells process a large number of conidia without internalization in this early time frame. We have also identified several host endocytosis markers that localize around internalized conidia as well as contribute to the processing of conidia. Understanding how these host endocytosis markers affect the processing of internal and/or external conidia may provide a novel avenue for therapeutic development.

## INTRODUCTION

Aspergillus fumigatus is an opportunistic fungal pathogen capable of invasive pulmonary infection in a diverse array of immunocompromised individuals (1). Chronic exposure to A. fumigatus may also result in the development and progression of an allergic response, the most severe being acute bronchopulmonary aspergillosis (ABPA). An allergic response occurs predominately in individuals with asthma or cystic fibrosis (2). A. fumigatus conidia are found abundantly throughout nature and the built environment, making exposure invariable. Most inhaled conidia are thought to be removed by mucociliary clearance in respiring organisms. However, A. fumigatus conidia can easily bypass mucociliary clearance and infiltrate lung alveoli due to their small size (2 to 3 μm in diameter). Airway epithelial cells (AECs) make up a large percentage of the pulmonary-air interface and are thought to play an important role in initial conidial recognition, processing, and immune signaling. There is a need to mechanistically understand how A. fumigatus conidia are processed by AECs (reviewed in references 3 and 4).

There has been substantial effort to characterize the interactions between A. fumigatus conidia and specific cell types of the respiratory epithelium (reviewed in reference 5). The bronchial epithelial layer is composed in part by ciliated, pseudostratified, columnar epithelial cells, as well as simple ciliated columnar or cuboidal epithelial cells (6). *In vitro* models of conidial uptake by the airway (bronchial) epithelium using the 16HBE14o- human bronchial epithelial cell line suggest 30 to 50% of conidia are internalized within 6 h (7). Studies by Richard et al. using immortalized human bronchial epithelial cells (BEAS-2B) indicate a very high internalization rate of conidia by BEAS-2B cells via an antibody-based epifluorescence microscopy assay designed to label only extracellular conidia (8). Botterel et al. observed a 20% internalization rate of conidia by primary nasal epithelial cells *in vitro* (9). Recently the paradigm of conidial internalization by bronchial airway epithelial cells has been challenged by Rammaert et al, as they did not observe internalization by bronchial epithelial cells during *in vivo* mouse models of A. fumigatus pulmonary infection using a novel bioimaging approach as well as transmission electron microscopy (10).

Adenocarcinomic human alveolar basal epithelial cells (A549) have been used extensively to study conidia internalized by nonprofessional phagocytic cells as well as the internalization of conidia by alveolar type II cells (type II pneumocytes). Type II pneumocytes are distinct from the airway (bronchial) epithelial cells as they lack cilia, are cuboidal, and are found specifically in alveoli (6). *In vitro* studies indicate A549 cells internalize 15 to 30% of encountered A. fumigatus conidia (11–13). Surface interactions between A. fumigatus and A549 cells occur in part through dectin-1, integrin α_5_β_1_, and E-cadherin (13–16). Subsequent internalization occurs through cofilin-1-, actin-, and phospholipase D-dependent mechanisms (13–16). Conidial internalization by A549 cells leads to lysosomal acidification and is associated with host endosomal/lysosomal markers LAMP1, CD63, and cathepsin D (13, 17). A. fumigatus proteins such as CalA mediate invasion of A549 cells in part by binding integrin α_5_β_1_ (18). Loss of CalA results in defective host cell invasion of alveoli, but not bronchi, during *in vivo* mouse models of pulmonary infection (18). Similarly, loss of the A. fumigatus pH response PacC transcription factor results in abnormal remodeling of the fungal cell wall during germination, culminating in diminished entry of germinating conidia into epithelial cells during mouse models of pulmonary infection and *in vitro* challenge assays using A549 cells (16). The rate, percentage, and mechanism of conidial internalization and killing by bronchial and alveolar epithelial cells are believed to be critical, as aberrations in these host processes may facilitate both immune evasion and disseminated infection.

We originally set out to determine the mechanism of conidial internalization and processing by bronchial airway epithelial cells using BEAS-2B cells as a model. Using confocal microscopy, we observed that many of the conidia in contact with cells appear to be internalized when a single plane is viewed. However, z-stack analysis clearly indicates the majority of these contacted conidia (80 to 90%) are not internalized, but are adherent on the apical side of BEAS-2B cells. Internalization of conidia was only observed for 10 to 20% of contacted conidia at 6 and 9 h postchallenge, but not at 3 h. Concurrent experiments using bone marrow-derived macrophages (BMDMs) resulted in the internalization of greater than 70% of contacted conidia within 1 h. This suggests internalization of conidia by BEAS-2B cells is not an immediate response and occurs for only a small percentage of the contacted conidial population. We also observed the processing of conidia by BEAS-2B cells *in vitro* is dependent on a number of factors, including the fungal isolate, the presence of serum, and the presence of liposomes to deliver small interfering RNA (siRNA). Our results highlight the discovery and importance of several cellular trafficking markers associated with processing and/or internalizing A. fumigatus conidia. These markers are comprised of caveolin, flotillin-2, RAB5C, RAB7A, RAB8B, 2xFYVE (a marker for phosphatidylinositol 3-phosphate [PI3P]), and FAPP1 (a marker for PI4P). Finally, we note that caution should be taken when using chemical inhibitors of endocytosis, as several inhibitors can impact fungal metabolic activity in the absence of BEAS-2B cells.

## RESULTS

### BEAS-2B cells internalize a small fraction of challenged conidia.

**FIG 1:**
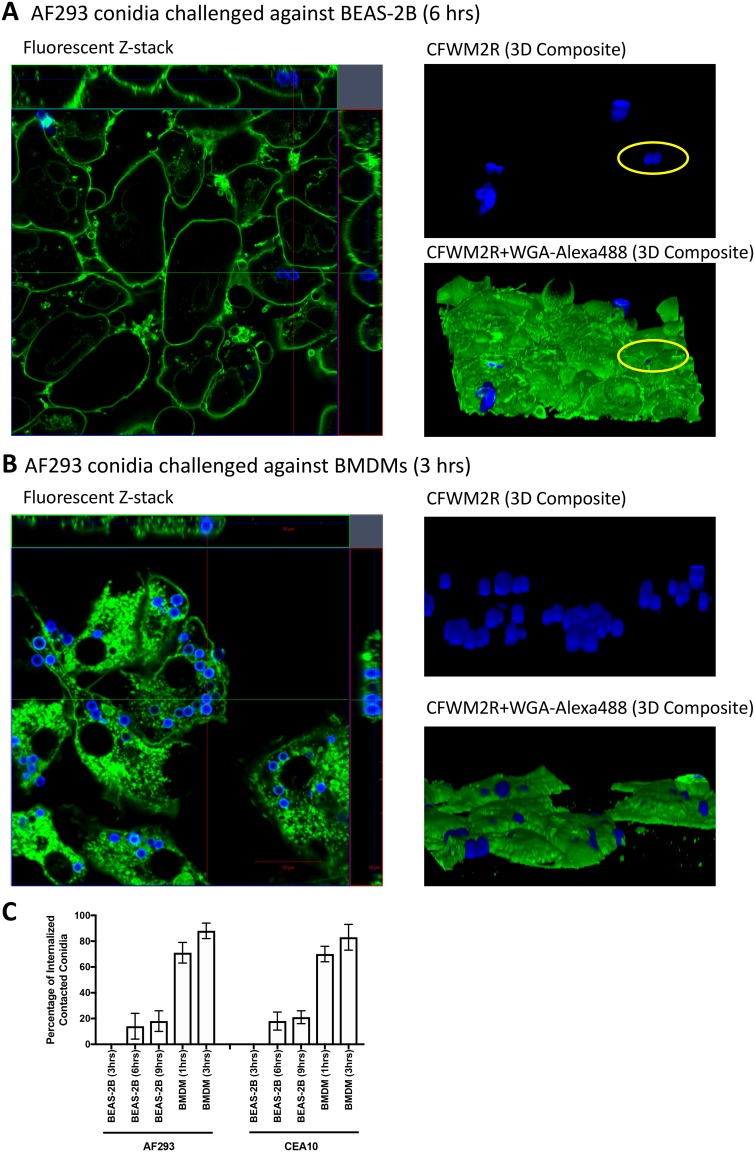
Confocal microscopy of conidial internalization by BEAS-2B cells and bone marrow-derived macrophages. Shown are representative z-stack and 3D composite images of conidial internalization by (A) BEAS-2B cells at 6 h postchallenge and (B) bone marrow-derived macrophages 3 h postchallenge. Conidia were labeled via calcofluor white M2R (CFWM2R) treatment prior to challenge. Cell membranes were labeled with wheat germ agglutinin conjugated to Alexa 488 (WGA-Alexa488) postfixation. A yellow circle in the 3D composite images of challenged BEAS-2B cells is a reference to show the position of internalized conidia. (C) Percentage of contacted conidia internalized by BEAS-2B cells at 3, 6, and 9 h postchallenge and bone marrow-derived macrophages 1 and 3 h postchallenge as determined by microscopy. A given experimental quantification looked at a minimum of 100 conidia and maximum of 200 conidia for each of the three challenges. Experiments were independently conducted 3 times (total, *n* = 9). Error bars indicate the standard error of the mean. See Movies S1 and S2 in the supplemental material.

We initially set out to determine if BEAS-2B cells, an immortalized cell line used as a model for bronchial airway epithelial cells, could internalize viable A. fumigatus conidia from the AF293 isolate. We concurrently challenged AF293 conidia against mouse bone marrow-derived macrophages (BMDMs) as they are a professional phagocytic cell type known to rapidly internalize and process conidia (12, 19). In order to precisely track conidia, we fluorescently labeled them with calcofluor white M2R (CFWM2R) prior to challenge. Conidia were then challenged against BEAS-2B cells for 3, 6, 9, and 12 h. Postchallenge and postfixation, we incubated cells with fluorescently tagged wheat germ agglutinin to label the host cell membrane. A composite 3D image was assembled from a z-stack spanning the entire volume of a given subgroup of cells. Based on these images, we only observed internalized conidia at 6 and 9 h postchallenge against BEAS-2B cells (Fig. 1A and C; see Movie S1 in the supplemental material). Of the conidia that were in contact with or adherent to BEAS-2B cells, only 10 to 20% of conidia were actually internalized when quantified (Fig. 1C). Similar percentages of internalization were observed for both the AF293 and CEA10 isolates (*P* > 0.10, Mann-Whitney U test [MWUT]) (Fig. 1C). It is important to note that a significant number of conidia were washed away during sample processing. Conversely, BMDMs internalized greater than 70% and 90% of contacted conidia within 1 h and 3 h postchallenge, respectively (Fig. 1B and C; see Movie S2 in the supplemental material). These experiments suggest that BEAS-2B cells are able to internalize conidia; however, BEAS-2B cells do not internalize a majority of contacted conidia like BMDMs. Interestingly, nearly all internalized conidia appeared to be swollen when imaged at more than 6 h postchallenge against BEAS-2B cells.

10.1128/mSphere.00663-18.8 MOVIE S1 Progression in a confocal z-stack of BEAS-2B cells challenged with AF293 conidia at 6 h postchallenge. The video begins at the proximal plane to the glass bottom well and concludes at the distal plane. Download Movie S1, MOV file, 19.2 MB. Copyright © 2019 Clark et al. 2019 Clark et al. This content is distributed under the terms of the Creative Commons Attribution 4.0 International license.

10.1128/mSphere.00663-18.9 MOVIE S2 Progression in a confocal z-stack of bone marrow-derived macrophages challenged with AF293 conidia at 3 h postchallenge. The video begins at the proximal plane to the glass bottom well and concludes at the distal plane. Download Movie S2, MOV file, 4.4 MB. Copyright © 2019 Clark et al. 2019 Clark et al. This content is distributed under the terms of the Creative Commons Attribution 4.0 International license.

### A flow cytometry assay to determine internalization and conidial metabolic activity.

Based on our confocal microscopy, we set out to develop a flow cytometry assay to determine the internalization and processing rates of viable, resting AF293 conidia by BEAS-2B cells. We challenged BEAS-2B cells with conidia for 3, 6, 9, and 12 h and then analyzed conidia through a flow cytometry-based assay for metabolic activity as well as calcofluor white M2R (CFWM2R) fluorescence (see Fig. S1 in the supplemental material). Immediately following challenge, CFWM2R was added to each well for 15 min to fluorescently label extracellular conidia; intracellular conidia are thought to be unlabeled. After CFWM2R labeling, medium was gently removed to avoid disturbance of extracellular conidia. Host cells were then lysed via NP-40 lysis buffer. Conidia were incubated for 1 h at 37°C in the presence of the metabolic marker FUN-1 to assess metabolic activity. Measurement of CFWM2R and FUN-1 fluorescence by conidia incubated in cell culture medium in the absence of cells was used to establish positive gating parameters for each time point (see Fig. S2 in the supplemental material). Greater than 70% of AF293 conidia were positive for FUN-1 metabolic activity at all measured time points when incubated in control medium (Fig. S2). CFWM2R fluorescent labeling of conidia was homogenous for 3 and 6 h postchallenge in control medium. At h 9 and 12 postchallenge, we observed a newly developed, very bright CFWM2R fluorescent population that was also FUN-1 positive in quadrant 2 of the flow plots (Fig. S2). We associated this population with the occurrence of germlings. The occurrence of short hyphae and elongating germlings was further verified through bright-field microscopy at 9 and 12 h postchallenge (see Fig. S3A to D in the supplemental material). Challenges against control medium were used to establish positive gating parameters for CFWM2R fluorescence and FUN-1 metabolic activity.

10.1128/mSphere.00663-18.2 FIG S1 Diagram of flow cytometry-based conidial challenge assay. (A) Conceptualization of the flow cytometry-based conidial challenge assay. Postchallenge, a subset of conidia will be internalized (quadrant 3 [Q3] and Q4), while another will be external (Q2 and Q1). Those that are internalized (Q3 and Q4) will have low labeling by calcofluor white M2R (CFWM2R). Likewise, a subset of conidia will be metabolically active and metabolize the FUN-1 dye, resulting in increased fluorescent intensity (Q3 and Q2) or inactivity (Q1 and Q4). (B) Postchallenge, cells are treated with CFWM2R for 15 min to presumably only label extracellular conidia. Postlabeling, medium is gently removed so as to not disturb conidia and cells are lysed using NP-40 cell lysis buffer. (C) Conidia are incubated in a solution of FUN-1 for 1 h at 37°C. Metabolically active conidia have a shift in fluorescence intensity in the FUN-1 channel. (D) The flow cytometry gating strategy is determined based on conidia incubated in medium in the absence of cells. Based on these gating strategies, the percentage of metabolically active conidia and conidia positive for CFWM2R fluorescence is determined for conidia challenged against BEAS-2B cells. Please note that in our study, we were unable to identify a clear bifurcation/separation for CFWM2R fluorescence and were therefore unable to use CFWM2R as a marker for internalization. Download FIG S1, PDF file, 0.1 MB. Copyright © 2019 Clark et al. 2019 Clark et al. This content is distributed under the terms of the Creative Commons Attribution 4.0 International license.

10.1128/mSphere.00663-18.3 FIG S2 Temporal analysis of CFWM2R fluorescence and FUN-1 metabolic activity by AF293 conidia postchallenge in control medium. (A and B) Representative flow plots of AF293 conidia (5 × 10^5^) incubated in control medium (A) containing or (B) lacking serum at 3, 6, 9, or 12 h postchallenge. (C) Representative histogram of CFWM2R fluorescence for conidia in the presence (S+) and absence (S−) of serum. Download FIG S2, PDF file, 0.4 MB. Copyright © 2019 Clark et al. 2019 Clark et al. This content is distributed under the terms of the Creative Commons Attribution 4.0 International license.

10.1128/mSphere.00663-18.4 FIG S3 Bright-field microscopy of AF293 conidial challenge assays. AF293 conidia (5 × 10^5^) were (A to D) incubated in control medium or (E to H) challenged against BEAS-2B cells at 3, 6, 9, or 12 h postchallenge. Download FIG S3, PDF file, 1.9 MB. Copyright © 2019 Clark et al. 2019 Clark et al. This content is distributed under the terms of the Creative Commons Attribution 4.0 International license.

**FIG 2:**
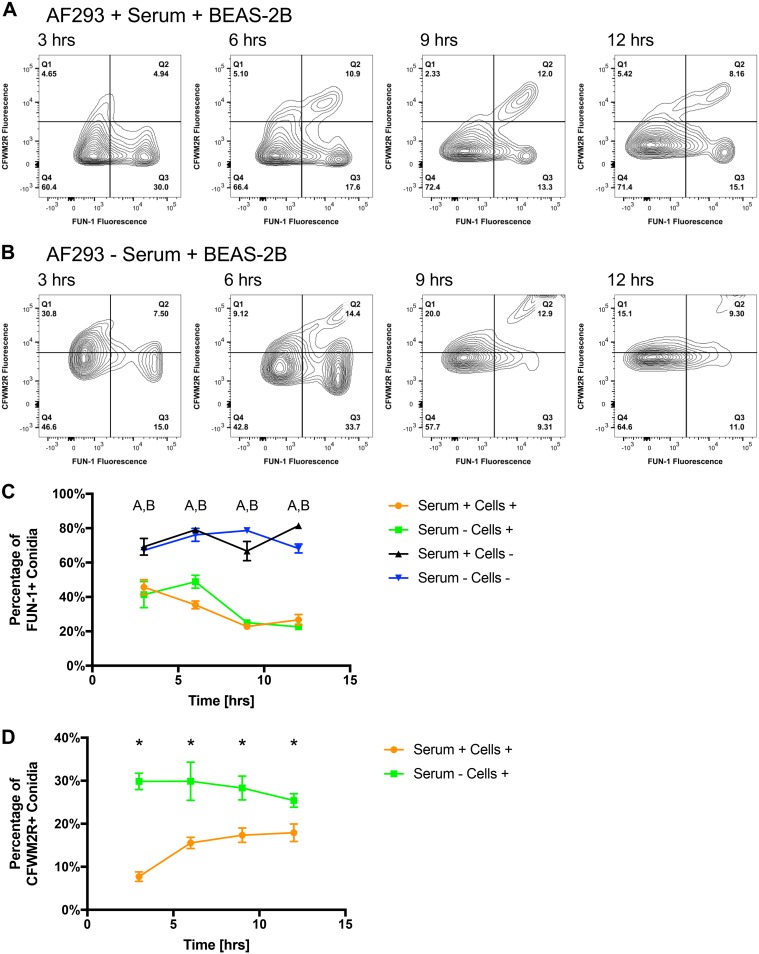
Temporal analysis of CFWM2R fluorescence and FUN-1 metabolic activity by AF293 conidia postchallenge against BEAS-2B airway epithelial cells. (A and B) Representative flow plots of AF293 conidia (5 × 10^5^) challenged against BEAS-2B cells in the (A) presence and (B) absence of serum at 3, 6, 9, or 12 h postchallenge. (C and D) Percentage of AF293 conidia positive for (C) FUN-1 or (D) CFWM2R fluorescence 3, 6, 9, or 12 h postchallenge against BEAS-2B cells in the presence and absence of BEAS-2B cells and serum. Experiments were independently repeated (total, *n* = 6). Statistical significance was determined using the Mann-Whitney U test. Error bars indicate the standard error of the mean. In panel C, “A, B” indicates statistical significance for FUN-1 fluorescence between cell-based challenges and medium-based challenges in the presence and in the absence of serum, respectively. In panel D, an asterisk indicates statistical significance for CFWM2R fluorescence between cell-based challenges containing versus lacking serum.

Based on the aforementioned control gating parameters, the overall percentage of FUN-1-positive conidia (consisting of populations that were both CFWM2R positive and negative) decreased from 45% at h 3, to 35% at h 6, to 25% at h 9 and 12 when challenged against BEAS-2B cells (Fig. 2A). A 40 to 70% decrease in conidia positive for FUN-1 metabolic activity was observed for conidia challenged against BEAS-2B cells in comparison to incubation in control medium for h 3 through 12 postchallenge (*P* < 0.05; MWUT) (Fig. 2C). Coupled with our confocal microscopy, these results indicate a method of A. fumigatus conidial processing by BEAS-2B cells that is independent of internalization within 3 h postchallenge. The decreased percentage of metabolically active conidia at 9 h postchallenge in comparison to 3 h postchallenge could be partially due to internalization, though other explanations exist.

### Lack of calcofluor white M2R fluorescence does not correlate with internalization.

We had thought to use CFWM2R as a way to selectively label extracellular conidia. Our rationale, and that of others, was that endocytosis by cells would provide a protective harbor for conidia so that they are shielded by CFWM2R labeling, antibody labeling, or a host-cell-impermeable antifungal (7, 8, 12, 13, 18). We utilized medium-only challenges to establish positive gating parameters, as conidia would be readily labeled by CFWM2R. Based on these parameters, approximately 6% of conidia were positive for CFWM2R staining (assumed to be extracellular and not internalized) by 3 h postchallenge against BEAS-2B cells (Fig. 2A and D). This value increased to 15 to 17% for h 6, 9, and 12, respectively. These results are in complete disagreement with what we observed via confocal microscopy and were more akin to the development of germinated conidia and short hyphae (Fig. S3E to H). Based on these findings, we conclude the variable CFWM2R fluorescence observed for conidia when challenged against BEAS-2B cells by flow cytometry could be due to both the amount of carbohydrate present on the conidia (state of germination) as well as conidia being adherent/internalized/partially internalized by cells. We suspect conidia are more than likely being killed or rendered metabolically inactive at various points during germination—hence the spectrum of CFWM2R fluorescence observed via flow cytometry without any clear bifurcation (Fig. 2A and B).

### Serum affects CFWM2R labeling, but not FUN-1 metabolic activity for AF293 conidia.

We set out to determine if serum impacted conidial processing and CFWM2R labeling as serum is known to affect a number of cellular processes. Conidia challenged in medium lacking serum had significantly increased CFWM2R fluorescence in comparison to conidia challenged in medium containing serum (Fig. S2B and C). This large increase in CFWM2R fluorescence in the absence of serum prompted us to develop different gating thresholds for conidia challenged against BEAS-2B cells in the absence of serum (Fig. 2B). Based on these thresholds, a significant increase in the percentage of conidia positive for CFWM2R fluorescence was noted at 3, 6, 9, and 12 h postchallenge against BEAS-2B cells in the absence of serum (*P* < 0.05; MWUT) (Fig. 2D). No statistical difference in metabolic activity was observed between the presence and absence of serum when conidia were challenged against BEAS-2B cells or in control medium (*P* > 0.10; MWUT) (Fig. 2C). We hypothesize that serum itself may affect CFWM2R labeling via masking of surface binding sites on conidia or components of serum may competitively bind CFWM2R.

### Heterogeneity in CFWM2R labeling and metabolic activity between CEA10 and AF293.

As heterogeneity among A. fumigatus isolates has become an evident theme (20, 21), we decided to determine if CEA10 conidia were as metabolically active as the AF293 isolate postchallenge with BEAS-2B cells. We used CEA10 conidia incubated in medium containing or lacking serum to set gating parameters for CFWM2R fluorescence as well as FUN-1 metabolic activity as there was again a notable difference in CFWM2R labeling due to serum (see Fig. S4 in the supplemental material). Approximately 100% of CEA10 conidia were metabolically active when incubated in control medium by 3 h in the presence or absence of serum (Fig. S4). The enhanced growth and germination of the CEA10 isolate were also evident in bright-field microscopy (see Fig. S5A and D in the supplemental material).

10.1128/mSphere.00663-18.5 FIG S4 Temporal analysis of CFWM2R fluorescence and FUN-1 metabolic activity by CEA10 conidia postchallenge in control medium. (A and B) Representative flow plots of AF293 conidia (5 × 10^5^) incubated in control medium (A) containing or (B) lacking serum at 3, 6, 9, or 12 h postchallenge. (C) Representative histogram of CFWM2R fluorescence for conidia in the presence (S+) and absence (S−) of serum. Download FIG S4, PDF file, 0.4 MB. Copyright © 2019 Clark et al. 2019 Clark et al. This content is distributed under the terms of the Creative Commons Attribution 4.0 International license.

10.1128/mSphere.00663-18.6 FIG S5 Bright-field microscopy of CEA10 conidial challenge assays. CEA10 conidia (5 × 10^5^) were (A to D) incubated in control medium or (E to H) challenged against BEAS-2B cells at 3, 6, 9, or 12 h postchallenge. Download FIG S5, PDF file, 1.8 MB. Copyright © 2019 Clark et al. 2019 Clark et al. This content is distributed under the terms of the Creative Commons Attribution 4.0 International license.

**FIG 3:**
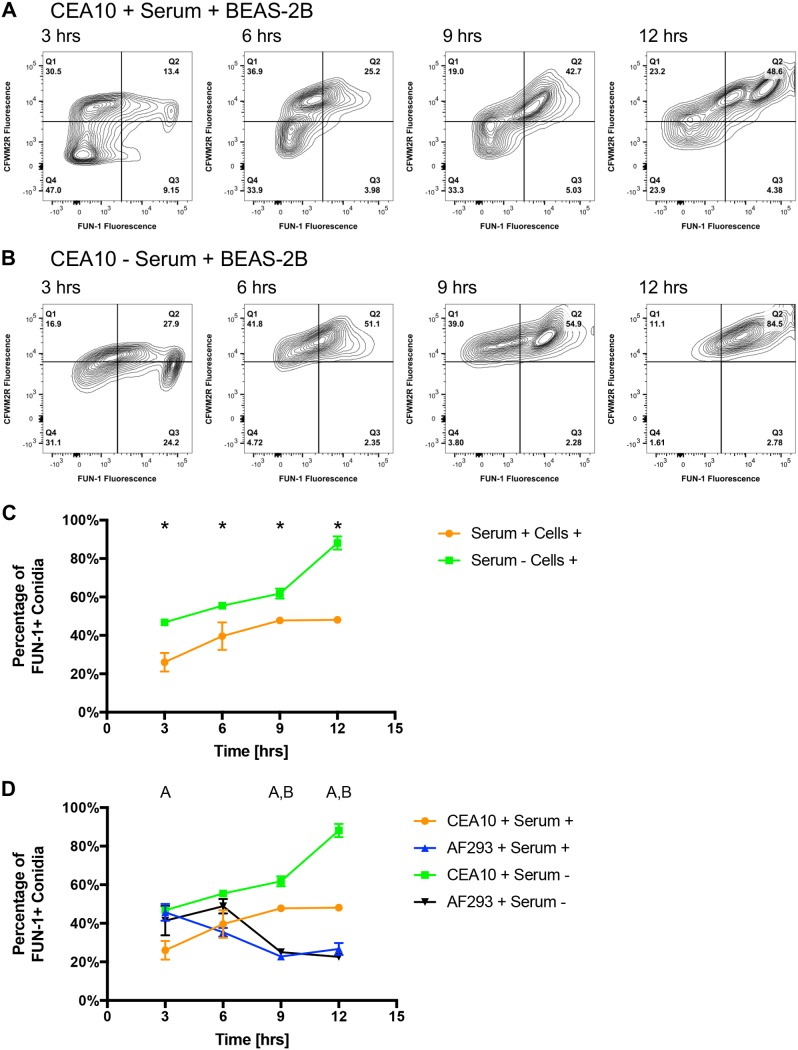
Temporal analysis of CFWM2R fluorescence and FUN-1 metabolic activity by CEA10 conidia postchallenge against BEAS-2B airway epithelial cells. (A and B) Representative flow plots of CEA10 conidia (5 × 10^5^) challenged against BEAS-2B cells in the (A) presence and (B) absence of serum at 3, 6, 9, or 12 h postchallenge. (C) Percentage of CEA10 conidia positive for FUN-1 fluorescence 3, 6, 9, or 12 h postchallenge against BEAS-2B cells in the presence and absence of serum. (D) Direct comparison of the percentages of CEA10 and AF293 conidia positive for FUN-1 fluorescence 3, 6, 9, or 12 h postchallenge against BEAS-2B cells in the presence and absence of serum. Experiments were independently repeated (total, *n* = 6). Statistical significance was determined using the Mann-Whitney U test. Error bars indicate the standard error of the mean. In panel C, an asterisk indicates statistical significance between cell-based challenges containing and lacking serum. In panel D, “A, B” indicates statistical significance between the percentages of metabolically active AF293 and CEA10 conidia challenged against BEAS-2B cells in the presence or absence of serum, respectively.

In the presence of serum, 25% of CEA10 conidia were metabolically active at 3 h postchallenge against BEAS-2B cells, and 45% were active by 9 and 12 h (Fig. 3A and C). In the absence of serum, 45% of CEA10 conidia were active by 3 h postchallenge against BEAS-2B cells and nearly 90% were active by 12 h (Fig. 3B and C). Direct comparison of FUN-1-positive conidia populations challenged against BEAS-2B cells indicates a larger percentage of FUN-1-positive conidia in the absence of serum than in the presence for all 4 time points (*P* < 0.05; MWUT) (Fig. 3C). A significantly higher percentage of metabolically active CEA10 conidia was also observed in comparison to AF293 when challenged against BEAS-2B cells at 9 and 12 h regardless of serum (*P* < 0.05; MWUT) (Fig. 3D). This large increase in CEA10 germinating conidia and elongating short hyphae in comparison to AF293 was also evident in the bright-field microscopy (Fig. S5E and H). Our findings indicate components of serum are beneficial for processing CEA10 conidia by BEAS-2B cells. These findings also indicate a much greater percentage of CEA10 conidia are metabolically active in comparison to AF293 postchallenge against BEAS-2B cells in the presence or absence of serum.

### Localization of endocytosis markers around internalized conidia.

**FIG 4:**
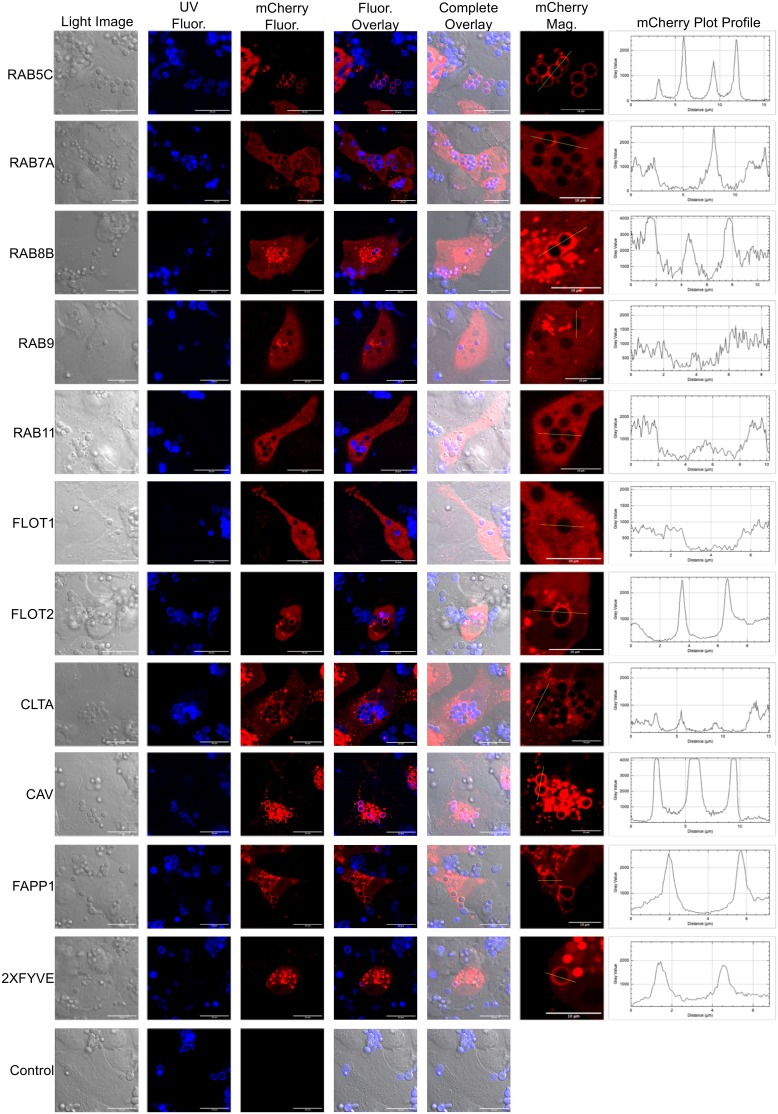
Localization of endosomal marker-mCherry fusion proteins with internalized A. fumigatus conidia. BEAS-2B cells were lipofected with a plasmid constitutively expressing an endosomal marker-mCherry chimera. Cells were challenged with A. fumigatus conidia (5 × 10^5^) that were stained with calcofluor white M2R (CFWM2R) and analyzed by confocal microscopy 9 h postchallenge. The UV channel shows CFWM2R-stained conidia. “mCherry Fluor” indicates fluorescence from endosomal marker-mCherry fusion protein. The fluorescent overlay is an overlay of the mCherry and UV channels. The complete overlay is an overlay of the light, UV, and mCherry channels. “mCherry Mag” shows a magnification of the region surrounding internalized conidia for mCherry signal. The mCherry plot profile shows the intensity of the mCherry signal that surrounds the conidia. Signal intensity corresponds to the yellow line in the mCherry Mag channel. FLOT1, flotillin-1; FLOT2, flotillin-2; CLTA, clathrin light-chain A; CAV, caveolin; FAPP1, PI4P-specific binding domain; 2xFYVE, PI3P-specific binding domain.

**FIG 5:**
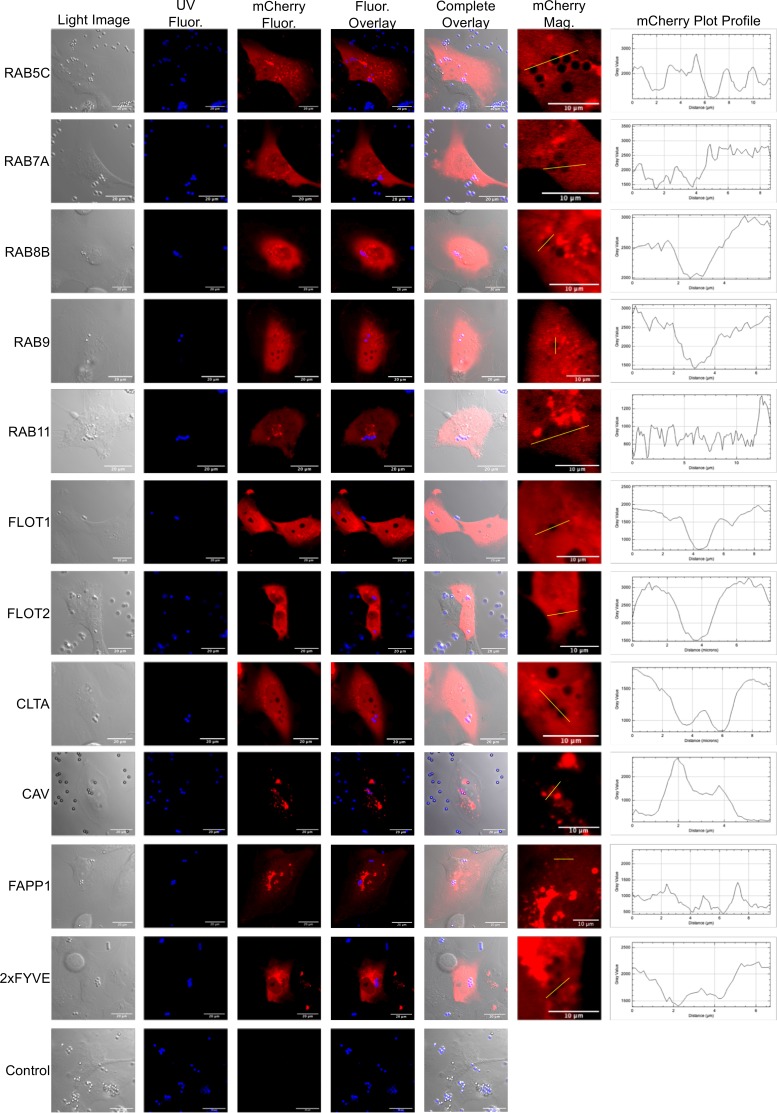
Localization of endosomal marker-mCherry fusion proteins with internalized fluorescent beads. BEAS-2B cells were lipofected with a plasmid constitutively expressing an endosomal marker-mCherry chimera. Cells were challenged with fluorescent beads (5 × 10^5^) that were analyzed by confocal microscopy 9 h postchallenge. The UV channel shows CWM2R-stained conidia. “mCherry Fluor” indicates fluorescence from the endosomal marker-mCherry fusion protein. The fluorescent overlay is an overlay of the mCherry and UV channels. The complete overlay is the overlay of the light, UV, and mCherry channels. “mCherry Mag” shows a magnification of the region surrounding internalized conidia specific for mCherry fluorescence. The mCherry plot profile shows the intensity of the mCherry signal that surrounds conidia. Signal intensity corresponds to the yellow line in the mCherry Mag channel. FLOT1, flotillin-1; FLOT2, flotillin-2; CLTA, clathrin light-chain A; CAV, caveolin; FAPP1, PI4P specific binding domain; 2xFYVE PI3P specific binding domain.

Fluorescently labeled host proteins produce a ring-like outline around internalized conidia (12, 13, 19). These images were our initial motivation to utilize microscopy as a means to dissect how conidia are trafficked once internalized by BEAS-2B. We transiently expressed 11 different cellular trafficking markers fused to mCherry in BEAS-2B cells and subsequently challenged these cells with viable, resting AF293 conidia or control fluorescent beads to determine if these markers specifically localized around internalized conidia (Fig. 4 and 5). A subset of these markers, flotillin-1, flotillin-2, clathrin, and caveolin, are associated with defined forms of initial endocytosis (22), while another subset, RAB5C, RAB7A, RAB8B, RAB9B, and RAB11, are associated with specific trafficking processes to various cellular compartments (23). A final subset of markers, the 2xFYVE domain and FAPP1 domain, are markers for the presence of phosphatidylinositol 3-phosphate (PI3P) and PI4P in cellular membranes, respectively (24–26). mCherry fusion proteins over expressed in BEAS-2B cells were shown to be intact and at the appropriate molecular weight via Western blot analysis (see Fig. S6 in the supplemental material). At approximately 9 h postchallenge, we were able to consistently visualize localization of flotillin-2, caveolin, RAB5C, RAB7A, RAB8B, FAPP1, and 2xFYVE-mCherry around internalized conidia (Fig. 4). Localization of fluorescent endosomal marker with conidia was specific for all markers except for caveolin and RAB5C, which also localized to internalized fluorescent beads with a weak signal intensity (Fig. 5). These results suggest caveolin and flotillin-2 are associated with internalized conidia and implicate trafficking to the early (RAB5C and 2xFYVE) and late (RAB7) endosomes. Localization of the PI4P biosensors FAPP1-mCherry and RAB8B-mCherry suggests the latter events occur through the *trans*-Golgi network (TGN) and/or vesicle transport to the plasma membrane.

10.1128/mSphere.00663-18.7 FIG S6 Western blot analysis of endosomal marker-mCherry fusion proteins transiently expressed in BEAS-2B cells. BEAS-2B cells were lipofected with a plasmid constitutively expressing an endosomal marker-mCherry chimera. Total protein from cells was analyzed 48 h postlipofection via Western blotting using an anti-His tag antibody. Download FIG S6, PDF file, 1.5 MB. Copyright © 2019 Clark et al. 2019 Clark et al. This content is distributed under the terms of the Creative Commons Attribution 4.0 International license.

**FIG 6:**
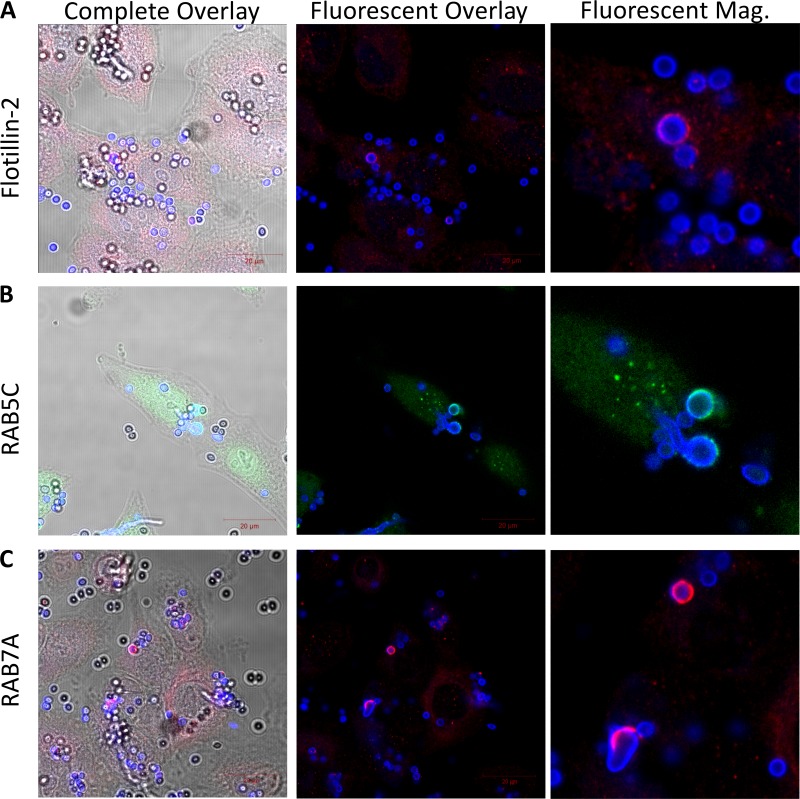
Immunofluorescent labeling of endogenous BEAS-2B host markers postchallenge with A. fumigatus conidia. BEAS-2B cells were challenged with A. fumigatus conidia for 9 h and fixed. Cells were probed using antibodies targeting endogenous (A) flotillin-2, (B) RAB5C (3), and (C) RAB7A and then subsequently probed using fluorescent secondary antibodies. The complete overlay is the overlay of the light, CFWM2R, and fluorescent antibody channels. The fluorescent overlay is the overlay of the fluorescent antibody and CFWM2R channels. “Fluorescent Mag” indicates magnification of the region surrounding internalized conidia specific for the fluorescent antibody and CFWM2R channels.

As transient overexpression may result in mislocalization of fluorescent fusion proteins, we performed immunofluorescence labeling of endogenous flotillin-2, RAB5C, and RAB7A in BEAS-2B cells challenged with AF293 conidia for 9 h. Fluorescent labeling of these markers indicates endogenous flotillin-2, RAB5C, and RAB7A do localize around internalized conidia (Fig. 6). We were unable to identify a suitable antibody targeting RAB8B or caveolin for these immunofluorescence labeling experiments.

### siRNA silencing of host endocytosis markers impacts metabolic activity of conidia.

**FIG 7:**
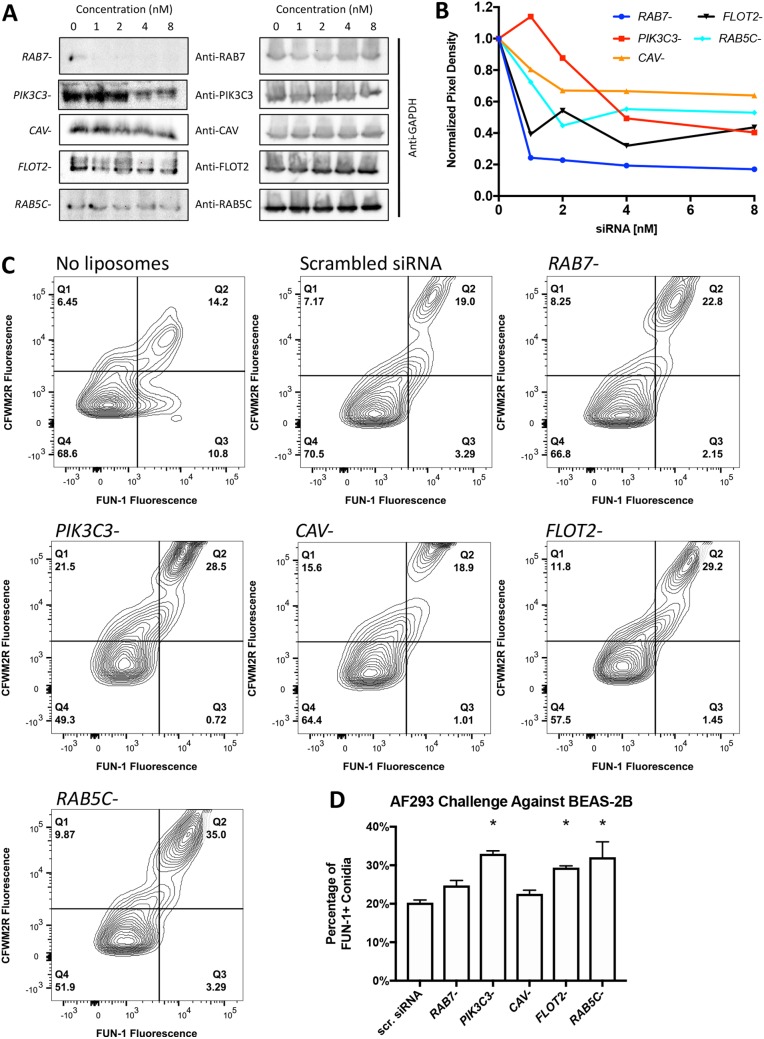
AF293 conidial challenge assays against BEAS-2B cells transiently silenced for an endosomal marker. (A and B) Efficacy of siRNA silencing on protein expression of a given endosomal marker via Western blotting. (B) Normalized pixel density of Western blot bands. The corresponding GAPDH signal density was used for normalization followed by 0 nM siRNA treatment. (C) Representative flow plots of conidia 9 h postchallenge against BEAS-2B cells silenced for a given endosomal marker. (D) Percentage of A. fumigatus conidia positive for FUN-1 fluorescence 9 h postchallenge against BEAS-2B cells silenced for a given endosomal marker. Experiments were independently repeated (total, *n* = 6). Statistical significance was determined using the Mann-Whitney U test. Error bars indicate the standard error of the mean. In panel D, an asterisk indicates statistical significance for FUN-1 fluorescence in the treatment group in comparison to BEAS-2B cells treated with scrambled siRNA.

To determine if caveolin, flotillin-2, RAB5C, RAB7A, and PIK3C3 (phosphatidylinositol 3-kinase catalytic subunit type 3, which is involved in the production of PI3P) impacted conidial metabolic activity, we transiently silenced the mRNA expression of a given gene via siRNA in BEAS-2B cells and subsequently challenged treated cells with viable, resting AF293 conidia (Fig. 7). Western blot analysis of total protein indicates transient siRNA was efficacious as there was a 30 to 80% reduction in protein levels for all targeted genes (Fig. 7A and B). A. fumigatus conidia challenged against BEAS-2B cells treated with liposomes produced a different CFWM2R and FUN-1 distribution in comparison to control cells (Fig. 7C). Specifically, the CFWM2R-negative and FUN-1-positive conidial population in quadrant 3 disappeared in flow cytometry plots when BEAS-2B cells were pretreated with liposomes. This suggested that liposomes themselves could alter the processing of conidia by BEAS-2B cells. Partial silencing of *PIK3C3*, *flotillin-2*, or *RAB5C* resulted in 29, 30, and 39% metabolically active conidia, respectively, in comparison to 22% observed in BEAS-2B cells treated with control liposomes combined with scrambled siRNA (Fig. 7D). This increase is quite notable as only 40% of conidia were metabolically active at 3 h postchallenge. An insignificant and marginal increase in metabolically active conidia was noted when caveolin (30% reduction in protein expression) or RAB7A (80% reduction in protein expression) was partially silenced.

### Small molecule inhibitors modulate CFWM2R fluorescence and metabolic activity.

**FIG 8:**
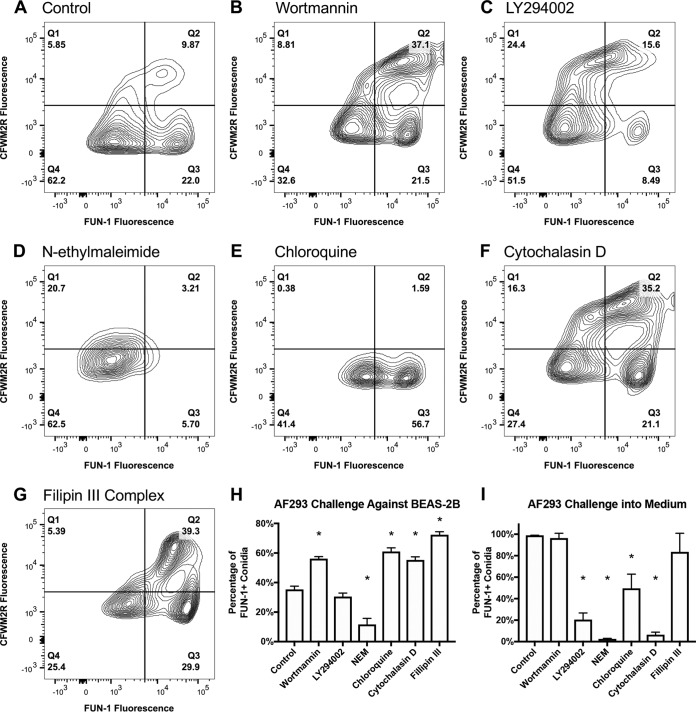
AF293 conidial challenge assays against BEAS-2B cells in the presence of chemical inhibitors of endocytosis. (A to G) Representative flow plots of AF293 conidia (5 × 10^5^) challenged against BEAS-2B cells at 9 h postchallenge pretreated with either wortmannin (1 μM), LY294002 (3 μM final concentration), *N*-ethylmaleimide (1 mM), chloroquine (100 μM), cytochalasin D (10 μM), or filipin III complex (50 μg/ml). (C) Percentage of A. fumigatus conidia positive for FUN-1 fluorescence 9 h postchallenge against BEAS-2B cells pretreated with a chemical inhibitor. (D) Percentage of A. fumigatus conidia positive for FUN-1 fluorescence 9 h postchallenge into control medium pretreated with a chemical inhibitor. Experiments were independently repeated (total, *n* = 6). Statistical significance was determined using the Mann-Whitney U test. Error bars indicate the standard error of the mean. In panels H and I, an asterisk indicates statistical significance for FUN-1 fluorescence in the treatment group in comparison to BEAS-2B challenges or incubation in medium lacking an inhibitor.

To further determine the role of PIK3C3 in conidial processing, we pretreated BEAS-2B cells with the irreversible PI3P kinase inhibitor wortmannin (1 μM final concentration) and the reversible inhibitor LY294002 (3 μM) for 3 h and subsequently challenged cells for 9 h (Fig. 8A to C). Analysis of conidia at 9 h postchallenge indicated a larger FUN-1-positive population when cells were pretreated with wortmannin (56%) in comparison to the control (35%) (*P* < 0.05; MWUT) (Fig. 8H).

We then tested a number of broad and specific small molecule inhibitors of endocytosis to determine if they could alter conidial processing by BEAS-2B cells. Pretreatment of cells for 3 h with *N*-ethylmaleimide (1 mM), a known inhibitor of clathrin-mediated endocytosis and stimulator of macropinocytosis (27), resulted in a tightly clustered CFWM2R-negative population of conidia and a notable decrease in metabolically active conidia (11%) in comparison to the control (35%) (*P* < 0.05, MWUT) (Fig. 8A, D, and H). Pretreatment of cells with chloroquine (100 μM), a known inhibitor of endosomal-lysosomal fusion and lysosome acidification (28), resulted in a conidial population that was 100% CFWM2R negative. Over 50% of conidia were metabolically active in comparison to the 35% observed in control challenges (*P* < 0.05; MWUT) (Fig. 8A, E, and H). Conversely pretreatment of cells with cytochalasin D (10 μM), an inhibitor of actin polymerization (29), resulted in an increased CFWM2R-positive population (44%) and an increased metabolically active population (55%) (*P* < 0.05; MWUT) (Fig. 8A, F, and H). Pretreatment of cells with filipin III complex (50 μg/ml), a potent binder of membrane cholesterol and disruptor of caveolae (30, 31), also resulted in an increased FUN-1-positive population (72%) (*P* < 0.05; MWUT) (Fig. 8A, G, and H).

Identical inhibitory experiments in the absence of BEAS-2B cells identified that a subset of inhibitors also directly impacted conidial metabolic activity (Fig. 8I). Pretreatment of control medium with LY294002, *N*-ethylmaleimide, or cytochalasin D prior to incubation with conidia resulted in near complete loss of conidial metabolic activity 9 h postincubation (*P* < 0.05; MWUT) (Fig. 8I). Pretreatment of medium with chloroquine resulted in an approximately 50% decrease in conidial metabolic activity (*P* < 0.05; MWUT) (Fig. 8I). The altered conidial metabolic activity and CFWM2R fluorescence observed in cell-based challenges in the presence of *N*-ethylmaleimide or chloroquine could also be attributed to the effect of those molecules on conidia themselves. Surprisingly, LY294002, a reversible inhibitor of PI3P kinase, had a considerable impact on conidia alone, suggesting a potential requirement for PI3P kinase function in conidial germination and metabolic function. Similar findings with cytochalasin D indicate actin polymerization is needed for A. fumigatus metabolic activity. The lack of effect of wortmannin and filipin III complex on conidial metabolic activity alone suggests their impact on BEAS-2B cells are specific to host PIK3C3 kinase and sequestration of membrane cholesterol, as well as disruption of caveolae.

## DISCUSSION

The interaction of A. fumigatus conidia with airway epithelial cells is known to result in important early immune signaling, as well as adhesion, internalization, and processing. The mechanism behind how conidia are internalized and/or killed by airway epithelial cells is not completely understood as many findings derive from *in vitro* studies involving alveolar A549 cells. We initially set out to gain insight into the kinetics and mechanism of conidial internalization and then aimed to generate a tentative cellular trafficking route of conidia by bronchial epithelial cells. Our results indicate a significant percentage of conidia are rendered metabolically inactive by BEAS-2B cells at 3 h postchallenge prior to internalization. Further, internalization was only observed for 10 to 20% of contacted conidia at 6 h postchallenge. The low percentage of internalization (10 to 20%) is similar to *in vitro* findings with A549 cells and primary nasal epithelial cells (9, 11–13). Experiments involving 16HBE14o- human bronchial epithelial cells, and BEAS-2B bronchial epithelial cells indicate a significantly higher internalization rate. An analysis of experimental conditions between studies suggests key differences in medium composition, fungal isolate, and method to measure cell entry (7, 8). Some direct comparisons between these studies may provide novel insight into how BEAS-2B cells can be modulated to process and internalize conidia.

Our results provide evidence that BEAS-2B cells process external conidia within 3 h postchallenge. We hypothesize that the rapid generation and secretion of antimicrobial peptides such as human β-defensin 2 may contribute to the early processing of extracellular conidia (32). The generation of reactive oxygen and/or nitrogen species may also function as a means to neutralize proximal conidia. Recently, Richard et al. found that BEAS-2B cells prevent germination of proximal conidia without internalization and suggest this occurs without the release of soluble compounds via a mechanism dependent on PI3P kinase (8). Rammaert et al. have been unable to identify internalization of conidia by bronchial airway epithelial cells in mouse models of invasive pulmonary aspergillosis, further proposing internalization is not one of the primary responses to contacted conidia (10). An important question has now arisen regarding the physiological relevance and occurrence of conidial internalization by airway epithelial cells. It will be important to determine how external conidia are processed by AECs as this appears to be a major route to neutralize conidia *in vitro*. It will also be interesting to explore why many of the internalized A. fumigatus conidia appear swollen and how this relates to immune evasion and processing.

Through this process, we have also identified a major deficiency in the use of CFWM2R as an indicator of internalization. We suspect CFWM2R functions as a marker for germination and potentially for adhesion. In our opinion, accurate internalization measurements can only be determined through the use of z-stacks, as a significant number of conidia thought to be internal via a single-plane analysis were actually external. The incorporation of an antibody-based approach utilized by others in the field in lieu of CFWM2R may provide further clarification (7, 18).

The variability among clinical and environmental isolates of Aspergillus fumigatus has emerged as an important theme. We observed striking differences between the CEA10 and AF293 clinical isolates in metabolic activity and germination postchallenge against BEAS-2B cells and even in control medium. Our results suggest CEA10 cells are able to germinate very rapidly postchallenge, and a significantly larger population of conidia are able to form hyphae and are metabolically active in comparison to the AF293 isolate. The CEA10 isolate has been previously shown to have greater fitness in low-oxygen environments in comparison to AF293, as well as enhanced virulence in mouse triamcinolone immunosuppression models of invasive pulmonary aspergillosis (20). The significantly enhanced *in vitro* growth and therefore generated biomass by the CEA10 isolate post-BEAS-2B challenge may explain why a heightened mortality is observed in steroid models of invasive pulmonary aspergillosis, as this model is thought to be driven in part by host immunopathogenesis in response to the fungus (33, 34).

Generally, receptor-mediated endocytosis occurs through a number of distinct or overlapping cytoplasmic adaptor proteins. These proteins are thought to facilitate specific biophysical properties of the internalizing membrane as well as mediate early stages of trafficking. We observed that both caveolin and flotillin-2 localize around internalized conidia. Endocytosis of Cryptococcus neoformans, a clinically relevant fungus, occurs through a caveolin-1-dependent process in human brain microvascular endothelial cells (35, 36). In contrast, host epithelial and endothelial cells internalize the hyphal form of clinically relevant dimorphic yeast Candida albicans through an interaction between C. albicans adhesin Als3 and host E-cadherin (epithelial cells) or N-cadherin (endothelial cells) (37). These interactions stimulate host actin rearrangement and require clathrin, dynamin, and cortactin for internalization (38). In response to β-glucans and zymosan, dectin-1 localizes to lipid raft domains for activation of phagocytosis and for cytokine signaling by dendritic cells (39). Interestingly, both A. fumigatus conidia and C. neoformans are internalized via flotillin-2 and caveolin-associated processes, while C. albicans hyphae are associated with clathrin and dynamin. It will be thought provoking to determine if A. fumigatus hyphae induce clathrin- and dynamin-mediated responses, suggesting a morphotype-specific response.

Postinternalization, vesicular bodies are rapidly modified for appropriate cellular trafficking by the early endosome. This conceptual trafficking hub can ultimately lead to a number of cellular organelles, including the *trans*-Golgi network (TGN), lysosome, or back to the plasma membrane. The trafficking to these organelles occurs through the coordinated functions of phosphoinositides, Rab, and Arf family proteins (40). RAB5 associates with vesicular bodies immediately after endocytosis and is essential for the recruitment of early effector proteins such as EEA1 and class III PIK3C3, thereby generating PI3P on the cytoplasmic side of the early endosome (41–43). EEA1 is critical for early endosome maturation through the fusion of early endosomes with late endosomes coated with RAB7A. This process frees RAB5, allowing late phagosomes to fuse with lysosomes to create phagolysosomes. Pathogenic bacteria, specifically Mycobacterium tuberculosis and Staphylococcus aureus, have the ability to blockade phagolysosome formation (44–47). Conversely, challenges of A. fumigatus conidia against macrophages result in rapid internalization and lysosome acidification through RAB7- and EEA1-associated pathways (19). We observed localization of RAB7A, RAB5C, and PI3P around internalized conidia, suggesting that conidia are processed via this route in BEAS-2B cells as well. siRNA silencing of *RAB5C* and *PIK3C3* resulted in a significantly increased metabolically active conidial population. Oddly, the effect of siRNA silencing of *RAB7A* on conidial processing was marginal, suggesting there may be redundancy in the conversion of early endosomes to late endosomes. It is important to reiterate that we were unable to determine if any of the increases in metabolically active conidia were internal or external to BEAS-2B cells when various endocytosis markers were silenced. Again, the use of *Aspergillus*-specific antibodies to differentiate between internal and external conidia would greatly aid in resolving this current quandary (7, 18).

Early endosomes coated in RAB5 and PI3P can also be shuttled into recycling vesicles and back to the plasma membrane through both slow and fast processes. Fast recycling occurs through RAB4-, RABb14-, and RAB15-mediated pathways, while slow recycling occurs in part through RAB11 (48). RAB11-mcherry did not localize around internalized conidia, suggesting this pathway is not utilized. Alternatively, early endosomes can mature into late endosomes and then be trafficked to the TGN, which is thought to be gradated with PI4P (49), via a RAB9-mediated pathway (50). We did observe the PI4P biosensor FAPP1-mCherry localize around conidia, suggesting conidia may localize to the TGN or components from the TGN may be shuttled to internalized conidia. The TGN can also be viewed as another conceptual trafficking hub as vesicles can then be shuttled to the endocytic recycling compartment via RAB11, the endoplasmic reticulum (ER) via RAB2, and back to the plasma membrane via RAB8A and/or RAB8B. We observed localization of RAB8B-mCherry around internalized conidia, but were unable to identify a suitable monoclonal antibody to validate localization for endogenous RAB8B. It will be essential to determine if RAB8B delivers components to proximal, adherent, and/or internalized conidia. The novel localizations of RAB8B and PI4P around internalized conidia will provide an avenue to dissect how the TGN may contribute to conidial processing.

Chemical inhibitors, though often fraught with secondary unintended effects, have been utilized to assess the modulation of endocytosis. We utilized a subset of chemical inhibitors—chloroquine, *N*-ethylmaleimide, cytochalasin D, and LY294002—that also impacted A. fumigatus conidial metabolic activity and germination in the absence of cells. Several inhibitors, such as chloroquine, have direct antifungal and antimalarial properties in addition to positively modulating cell-mediated responses (51–54). Similarly, we show LY294002, a known reversible inhibitor of PIK3C3, also functions in inhibiting A. fumigatus metabolic activity in the absence of cells. These findings suggest PI3P kinase activity is important for fungal activity and may provide a new avenue to develop antifungals targeting PI3P kinases. The results of cholesterol sequestration via filipin III complex are in agreement with the localization of flotillin-2 as it is commonly found in lipid rafts rich in cholesterol (55). Again, we were unable to ascertain if the effects of wortmannin or filipin III complex on BEAS-2B cells were impacting internal and/or external conidia. Richard et al. suggests that LY294002 inhibition of PIK3C3 kinase specifically impacts external conidia (8). An exciting future prospect is the analysis of LY294002 analogs to identify small molecules that specifically target A. fumigatus PI3P kinases and not mammalian PI3P kinases.

Cellular internalization and trafficking of conidia present an important yet technically challenging area of microbe-host interactions. Understanding the mechanism behind how AECs and phagocytic cells engulf and process conidia offers a novel opportunity to design host-targeted therapeutics to aid in early stages of clearance and processing. It will be exciting to determine if conidial internalization and processing by professional phagocytic cells occur via the newly identified flotillin-2-, caveolin-, and RAB8B-dependent pathways and whether the process involves PI4P. Determining the physiological occurrence of conidial internalization by AECs in healthy and immunosuppressed hosts may reveal the relevance of this process. How AECs process conidia in the absence of internalization, and if they utilize any of the identified pathways to deliver defense-related compounds, will be of novel significance.

## MATERIALS AND METHODS

### Plasmid preparation.

Genes were synthesized with flanking AttB1 and AttB2 gateway cloning sites into pUC57. Genes were recombined into a pDONR201 entry vector and then a destination vector based upon a modified pcDNA3.1 vector containing AttR sites encompassing a chloramphenicol resistance cassette and a ccdB cassette. This gateway cassette was followed by a sequence encoding the fluorescent reporter mCherry and a C-terminal 6-histidine tag (see Text S1 in the supplemental material).

10.1128/mSphere.00663-18.1 TEXT S1 List of synthetic DNA sequences utilized for this study followed by the predicted mCherry fusion protein sequence. Download Text S1, DOCX file, 0.1 MB. Copyright © 2019 Clark et al. 2019 Clark et al. This content is distributed under the terms of the Creative Commons Attribution 4.0 International license.

### Culturing and lipofection of BEAS-2B cells.

Human bronchial epithelial cells (BEAS-2B; ATCC CRL-9609) were routinely cultured in RPMI base medium supplemented with 10% fetal bovine serum (FBS) and 1× penicillin-streptomycin in 75-cm^2^ tissue culture flasks at 37°C in the presence of 5% CO_2_. Cells were grown to approximately 75% confluence and passaged. Cells were discarded after their 9th passage. Cells were plated in a 24-well plate for all assays at a density of 250,000 cells per well. Cells were allowed to grow for approximately 48 h prior to challenge with a medium exchange at 24 h prior to challenge. Serum-starved cells were given RPMI medium lacking FBS 24 h prior to challenge. Transient expression of mCherry fusion proteins occurred through a standardized lipofection-based protocol described by Clark et al. (56). Transient silencing of a specified gene occurred through Lipofectamine LTX with Plus solution via the manufacturer's protocol. Specifically, liposomes were prepared using 1 to 5 μl siRNA (Santa Cruz), 5 μl Plus solution per well, and 5 μl Lipofectamine LTX per well.

### Extraction and culturing of bone marrow-derived macrophages.

Bone marrow-derived macrophages were extracted from male mice between 20 and 30 weeks of age. Extraction of BMDMs was conducted as previously described by Zhang et al. (57). All animal studies were carried out in strict accordance with the recommendations in the *Guide for the Care and Use of Laboratory Animals* (58). All protocols involving animals were approved by the Institutional Animal Care and Use Committee at Virginia Tech and in compliance with Public Health Service Policy (PHS [approval no. 16-085]). BMDMs were seeded at a density of 500,000 cells per well in a 24-well glass bottom plate 6 days postdifferentiation. Postchallenge, cells were fixed in ice-cold methanol (chilled at −20°C) for 15 min at room temperature and washed three times with phosphate-buffered saline (PBS) for 5 min. Cells were then incubated with wheat-germ agglutinin conjugated to Alexa 488 (5 μg/ml) in Hanks’ balanced salt solution for 30 min and washed three times with PBS for 5 min. Cells were then imaged by confocal microscopy. Similar methods were used for BEAS-2B cells challenged with A. fumigatus conidia.

### Immunofluorescence labeling.

Cells were fixed in ice-cold methanol (chilled at −20°C) for 15 min at room temperature and washed three times with PBS for 5 min. Cells were then permeabilized using PBS plus 0.1% Triton X-100 and washed three times with PBS for 5 min. Cells were placed in a blocking solution (1% bovine serum albumin [BSA] in PBS plus 0.1% Tween 20 [PBS-T]) for 1 h at room temperature Cells were then incubated in blocking solution with primary antibody (1:200) overnight at 4°C and washed three times with PBS. Cells were then incubated in blocking solution containing fluorescent secondary antibody (1:200) in the dark for 1 to 2 h and washed three times with PBS in the dark. Cells were then imaged by confocal microscopy. The primary antibodies are described below. The following fluorescent secondary antibodies were used: NL493 anti-goat IgG and NL637 anti-rabbit IgG.

### Microscopy.

All imaging was conducted using the multitrack feature of a Zeiss LSM 510 laser scanning confocal microscope. Control polystyrene beads and calcofluor white M2R were excited using a 25-mW Enterprise 353 laser (10%) and captured using a 385- to 470-nm broad band filter. Detector gains were ∼1,100 for conidia and ∼400 for beads, with an amplifier offset of approximately −0.5. mCherry fusion proteins were excited using a 1-mW 543 HeNe laser (100%), and emission was captured using a 560- to 615-nm broad band filter. Detector gain was ∼750, with an amplifier offset of approximately −0.1. All images were recorded as 1,024 by 1,024 8-bit format. Images are an average of 8 scans with a pixel dwell speed ranging from 0.5 to 1.0 μs.

### Western blotting.

Approximately 48 h postlipofection, cells were washed twice in 1 ml of Dulbecco’s phosphate-buffered saline (DPBS) and lysed using 250 μl of NP-40 lysis buffer containing protease inhibitor. Samples were immediately mixed with 50 μl of 6× protein loading buffer and flash frozen in liquid nitrogen. Whole-cell aliquots were boiled for 5 min and separated by 7.5% SDS-PAGE. Samples were transferred to Hybond-C Extra nitrocellulose membrane using a Bio-Rad wet transfer system. Membranes were blocked for 1 h at room temperature using 20 ml of PBS-T (0.1% Tween 20) with 3% BSA. Primary antibody (1:10,000) was added to fresh blocking buffer overnight at 4°C. Membranes were washed 3 times with PBS-T for 10 min at room temperature Membranes were probed with secondary antibody (1:20,000) in PBS-T for 1 h at room temperature Membranes were subsequently washed 3 times with 20 ml PBS-T for 10 min at room temperature Blots were imaged using chemiluminescent substrate and visualized using a ChemiDoc XRS+ work station with exposures from 10 s to 10 min in 10-s increments. Quantification of Western blot bands was done via Image Lab using the band analysis tool (adjusted volume). Band intensity was first normalized by the corresponding GAPDH (glyceraldehyde-3-phosphate dehydrogenase) band intensity and then the control-only liposome treatment. The following antibodies were utilized for this study: from Santa Cruz Biotechnology, Inc., caveolin-1, sc-894; flotillin-2, sc-25507; PIK3C3 p100, sc-134986; RAB5C, sc-26570; RAB7, sc-10767; from ICL Lab, anti-6× His, RHIS-45A-Z; and from GE Life Sciences, anti-rabbit IgG-horseradish peroxidase (HRP), NA9340V.

### Cell-line-based conidial challenge assays.

Aspergillus fumigatus AF293 and CEA10 were grown on glucose minimal medium (GMM) plates at 37°C for approximately 4 to 6 days. Conidia were harvested in 20 ml of PBS-T and passed through cheesecloth to remove any residual hyphae. Conidia were diluted to a concentration of 5 × 10^5^ in 50 μl of PBS-T. Approximately 5 × 10^5^ conidia were added per well in a 24-well plate. Challenged cells were immediately spun for 5 min at 1,000 × *g* at room temperature to synchronize and maximize contact. Plates were then stored at 37°C in the presence of 5% CO_2_ for approximately 3, 6, 9, or 12 h. At the conclusion of a challenge, calcofluor white M2R was added (CFWM2R; 25 μM) and incubated with cells for 15 min at 37°C with 5% CO_2_. Supernatants were gently removed, and cells were resuspended in 250 μl NP-40 lysis buffer supplemented with FUN-1 metabolic dye (2.5 μM) for 1 h at 37°C in the presence of 5% CO_2_. Conidia were pelleted and then resuspended in 500 μl of fluorescence-activated cell sorter (FACS) buffer and immediately assessed by flow cytometry. Fungal conidia (50,000) and short hyphae from independent challenges were analyzed by flow cytometry for FUN-1 and CRWM2R fluorescence. Positive gating was determined using conidial challenge on empty tissue culture wells containing the respective cell culture medium as well as just cell controls. Independent experiments were run in triplicate. All experiments were independently repeated (total, *n* = 6). Statistical significance was determined using the Mann-Whitney U test.

For studies involving chemical inhibitors, cells or medium was pretreated with a given inhibitor for 3 h prior to challenge. The following inhibitors were used at the stated final concentrations (in parentheses): LY294002 (3 μM), wortmannin (1 μM), *N*-ethylmaleimide (1 mM), cytochalasin D (10 μM), filipin III complex (50 μg/ml), and chloroquine (100 μM).
