# Supplementary figures and images for: Endocytic Markers Associated with the Internalization and Processing of Aspergillus fumigatus Conidia by BEAS-2B Cells
Source: mSphere. 2019 Feb 6;4(1):e00663-18. doi: 10.1128/mSphere.00663-18 (PMC6365614; doi:10.1128/mSphere.00663-18)

A

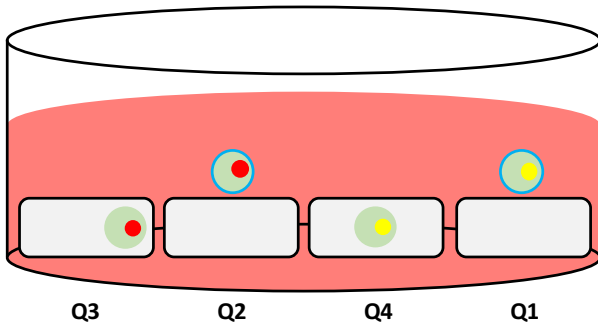

B

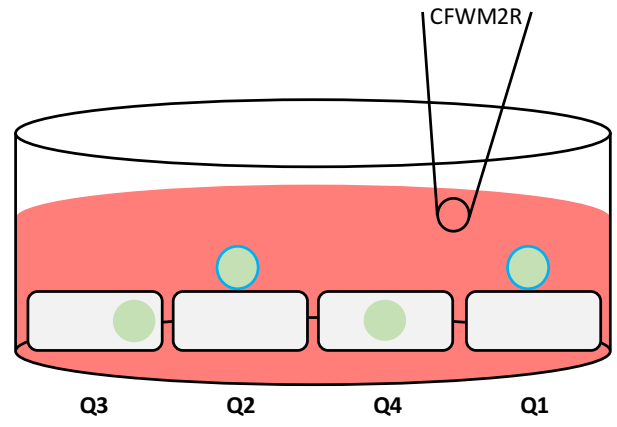

C

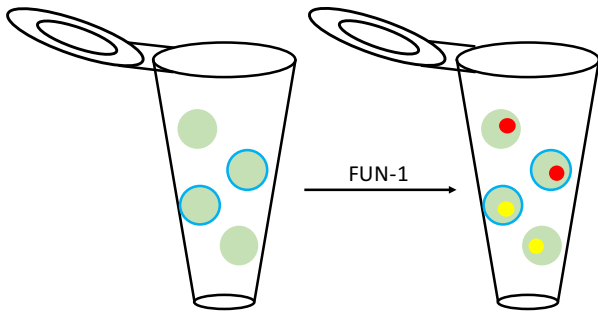

D

| CFWM2R | Q1                      | Q2                      |
|--------|-------------------------|-------------------------|
|        | FUN-1 (-)<br>CFWM2R (+) | FUN-1 (+)<br>CFWM2R (+) |
|        | FUN-1 (-)<br>CFWM2R (-) | FUN-1 (+)<br>CFWM2R (-) |
|        | Q4                      | Q3                      |
| FUN-1  |                         |                         |

Supplement: FIG S1 [file mSphere.00663-18-sf001.pdf]

## A AF293

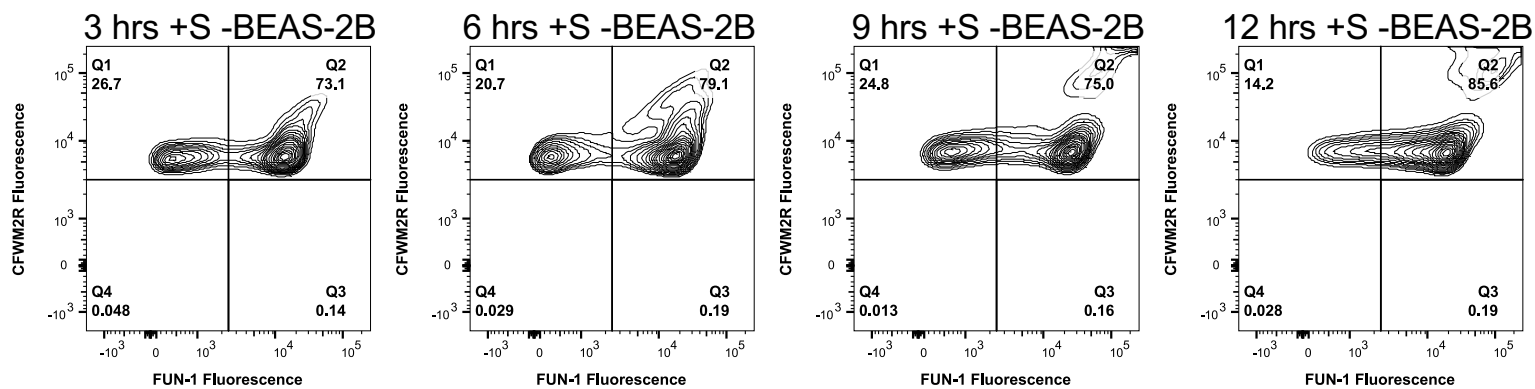

## B AF293

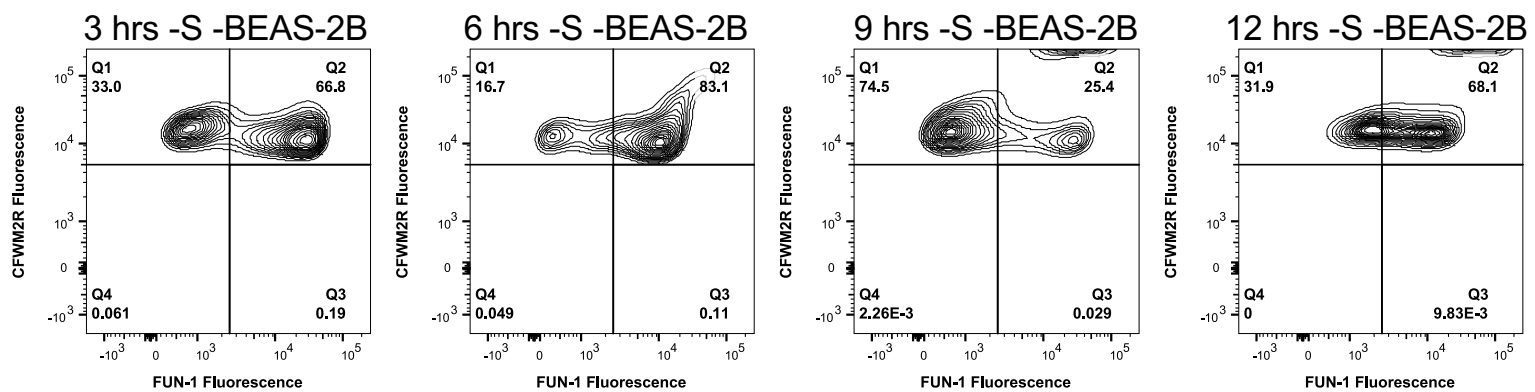

## C

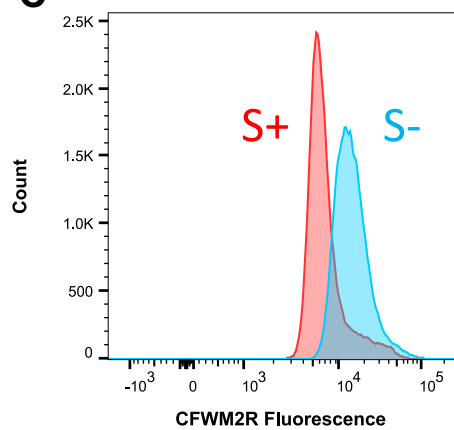

Supplement: FIG S2 [file mSphere.00663-18-sf002.pdf]

**A**

3 hrs- AF293

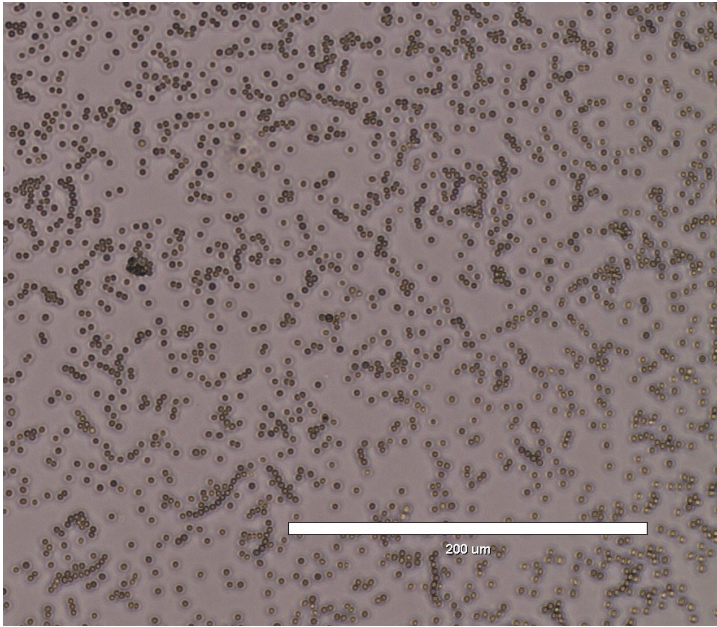**B**

6 hrs- AF293

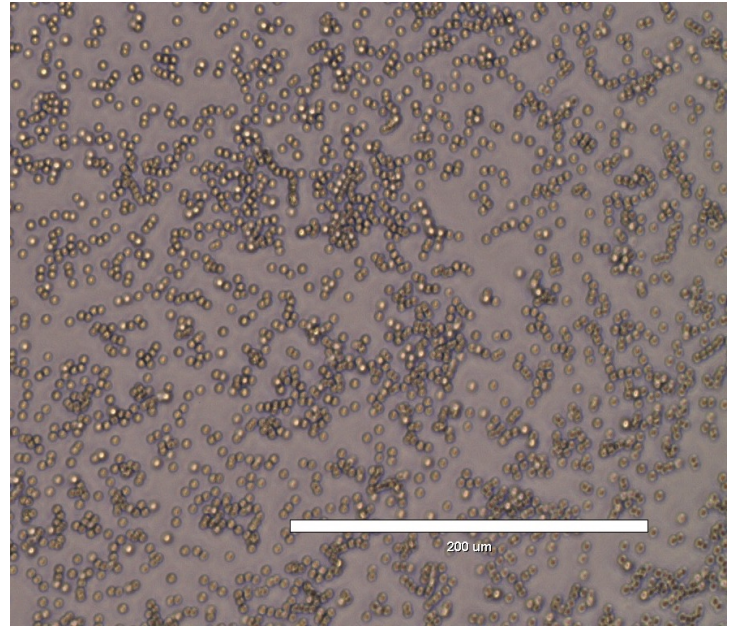**C**

9 hrs-AF293

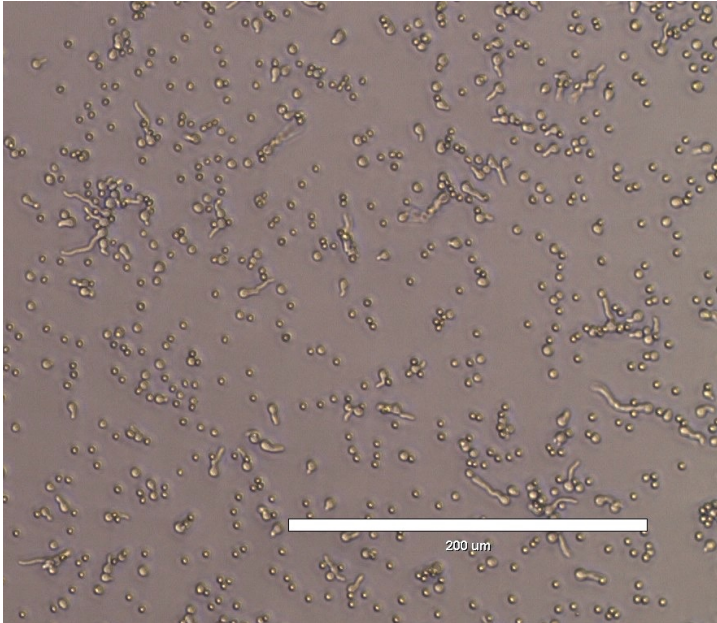**D**

12 hrs-AF293

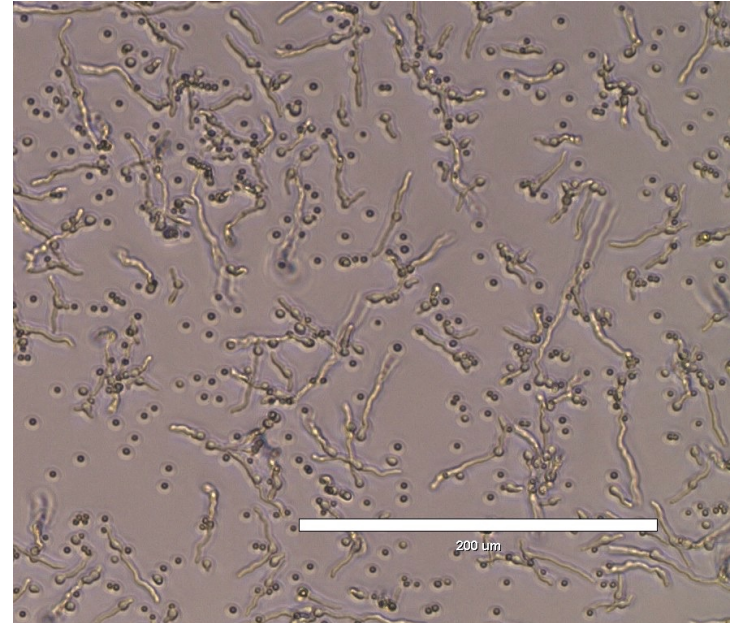

**E** 3 hrs- AF293 + BEAS-2B

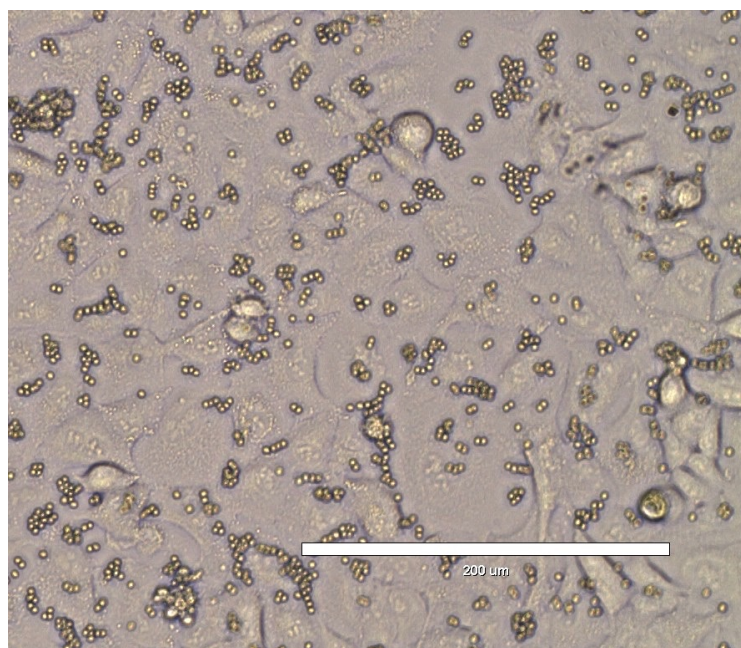

**F** 6 hrs- AF293 + BEAS-2B

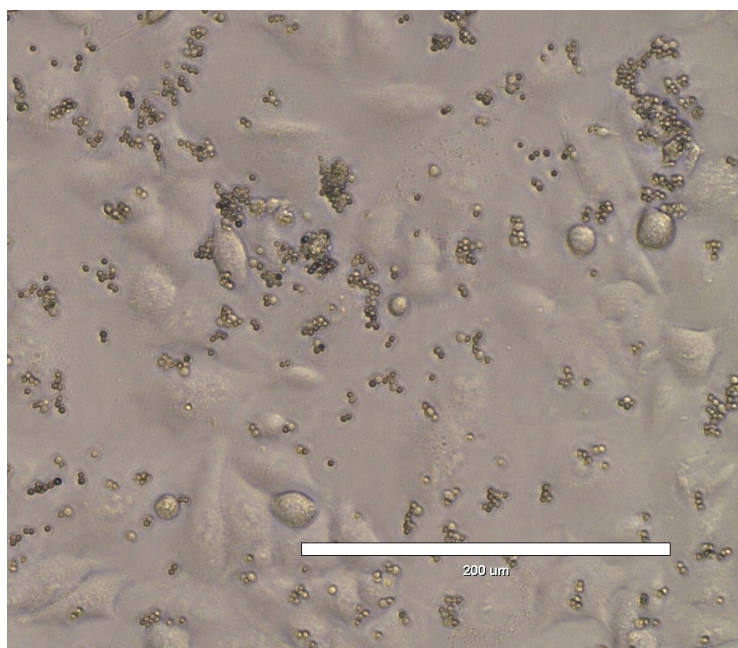

**G** 9 hrs-AF293 + BEAS-2B

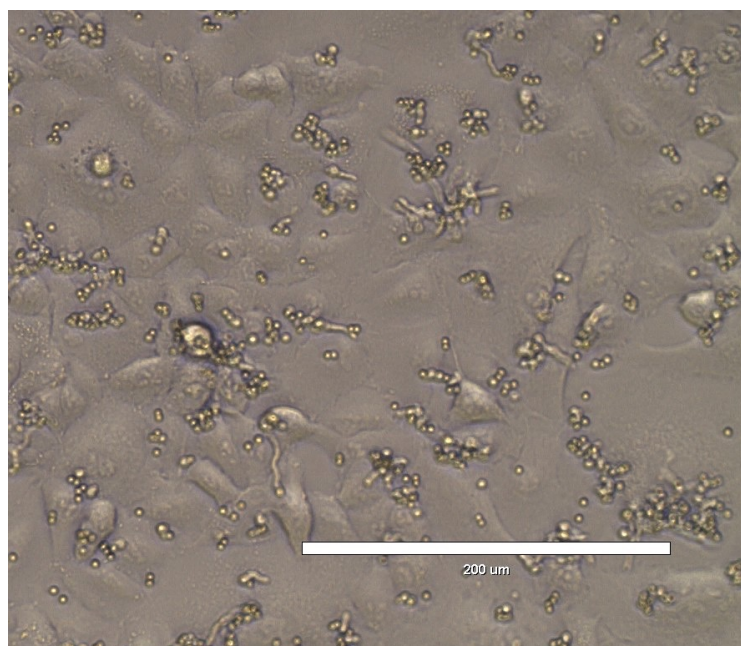

**H** 12 hrs-AF293 + BEAS-2B

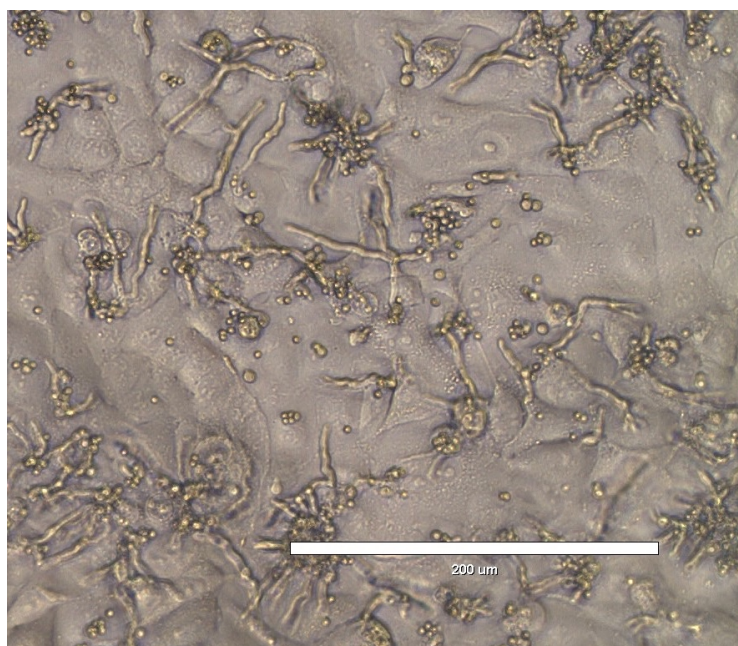

Supplement: FIG S3 [file mSphere.00663-18-sf003.pdf]

## A CEA10

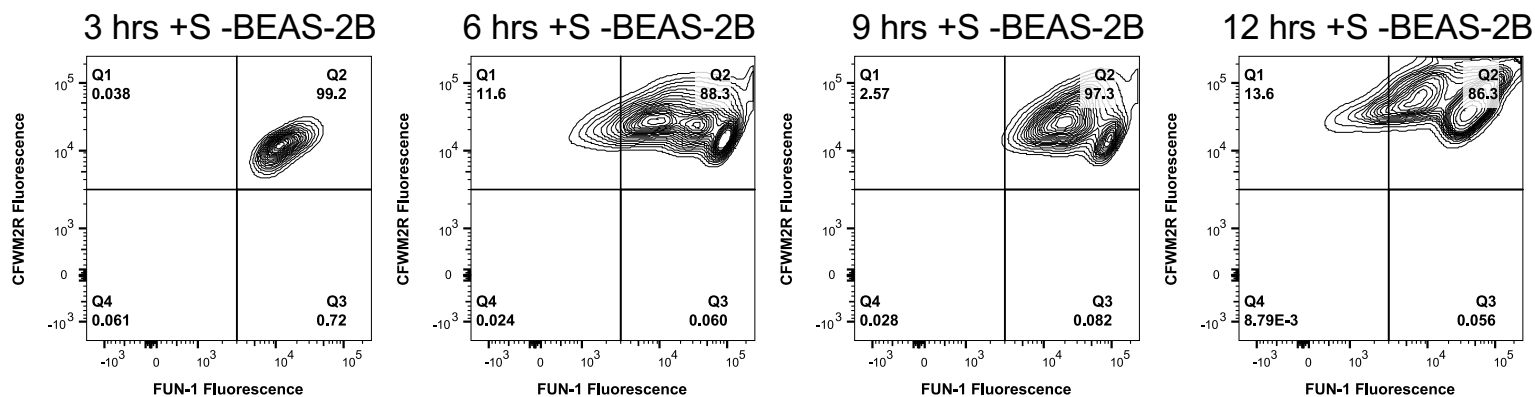

## B CEA10

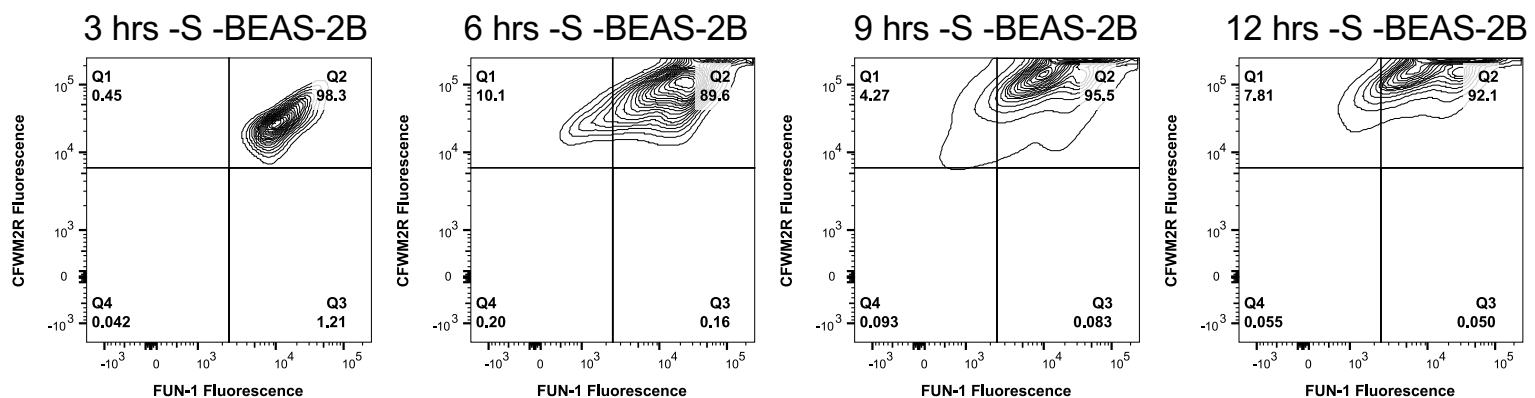

## C

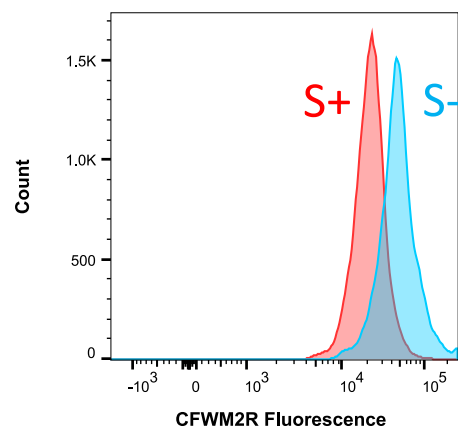

Supplement: FIG S4 [file mSphere.00663-18-sf004.pdf]

**A** 3 hrs- CEA10

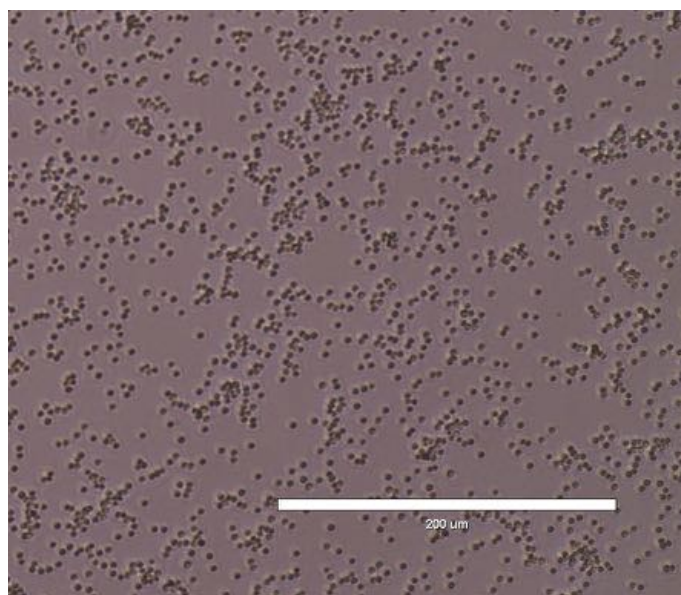

**B** 6 hrs- CEA10

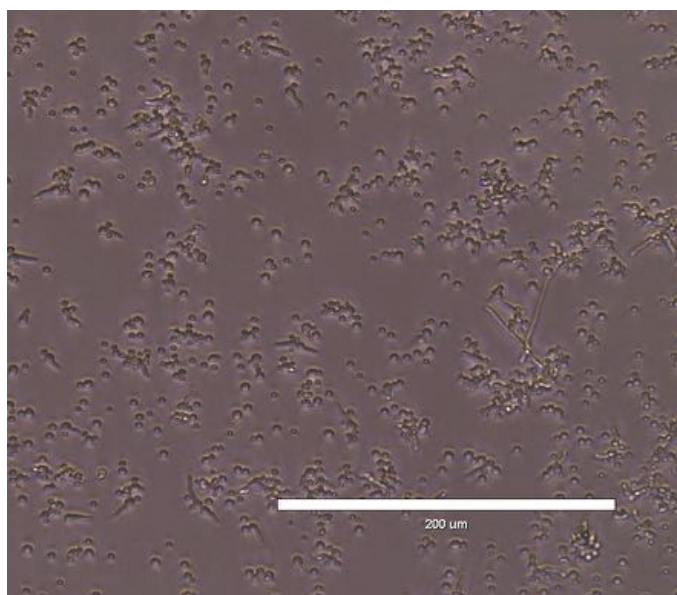

**C** 9 hrs- CEA10

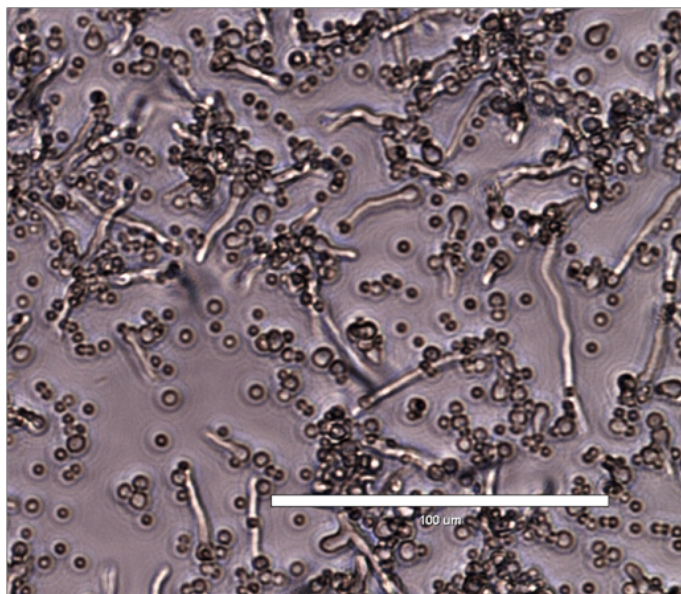

**D** 12 hrs- CEA10

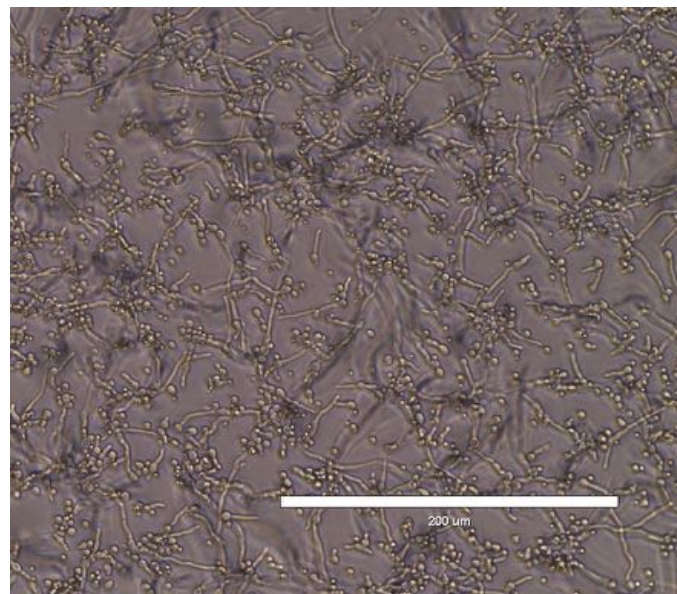

**E** 3 hrs- CEA10 + BEAS-2B

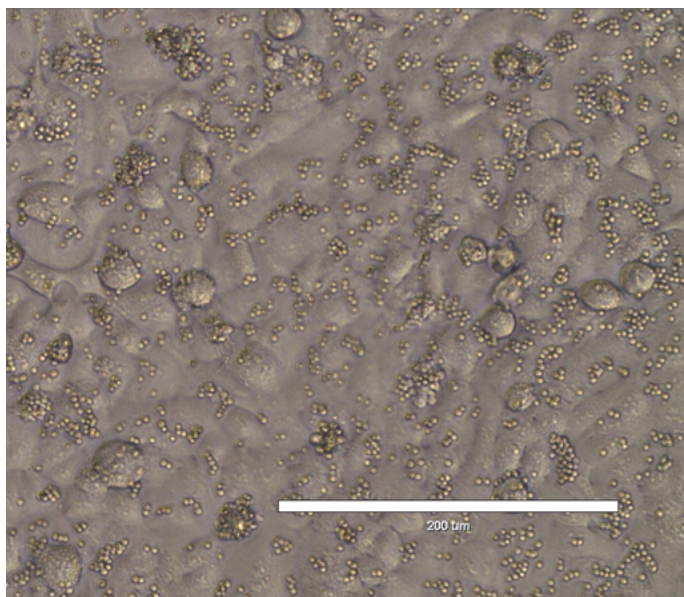

**F** 6 hrs- CEA10 + BEAS-2B

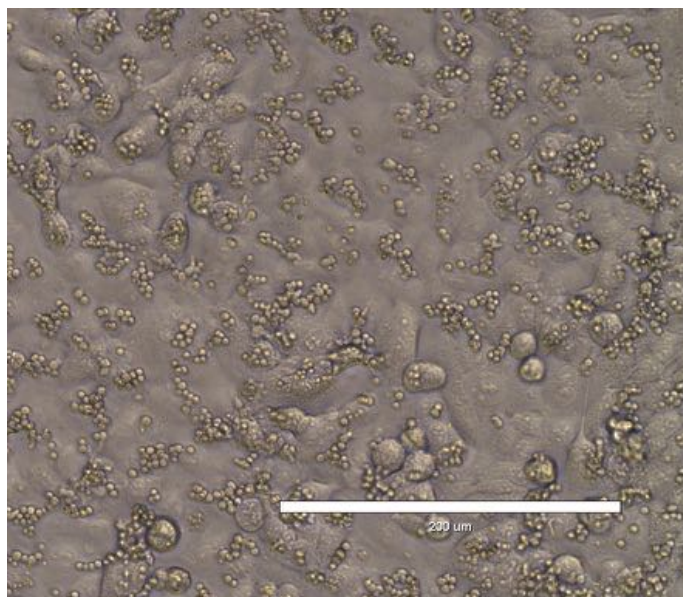

**G** 9 hrs- CEA10 + BEAS-2B

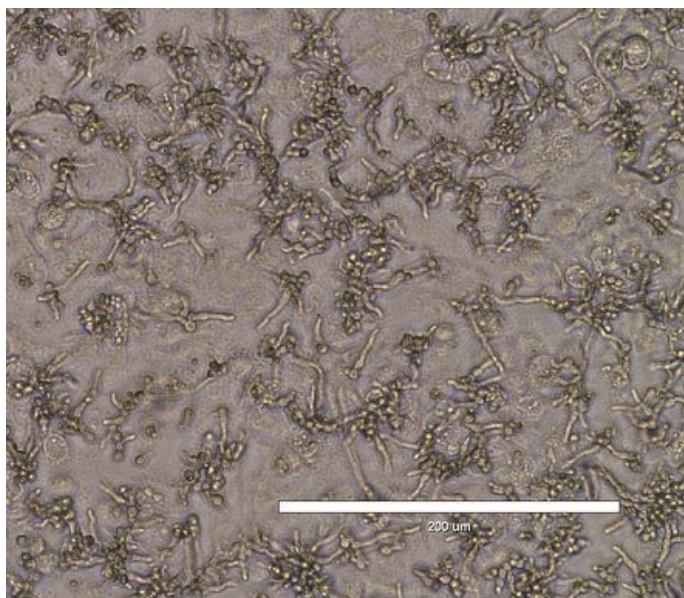

**H** 12 hrs- CEA10 + BEAS-2B

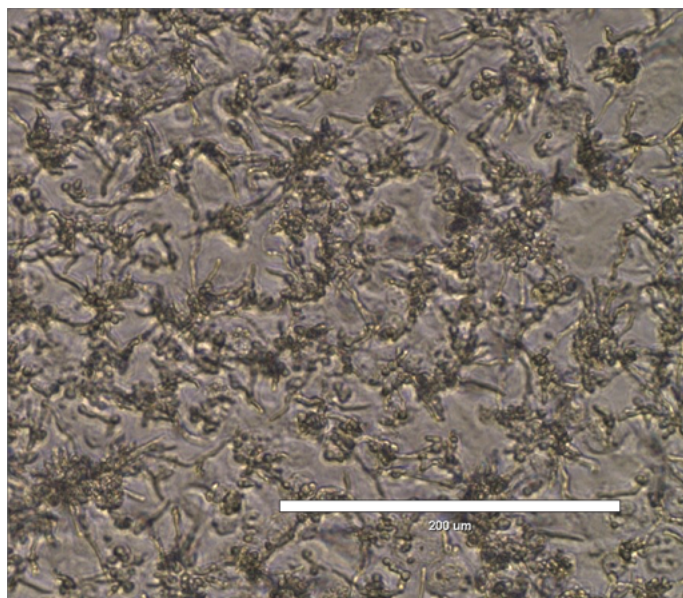

Supplement: FIG S5 [file mSphere.00663-18-sf005.pdf]

Ponseau S

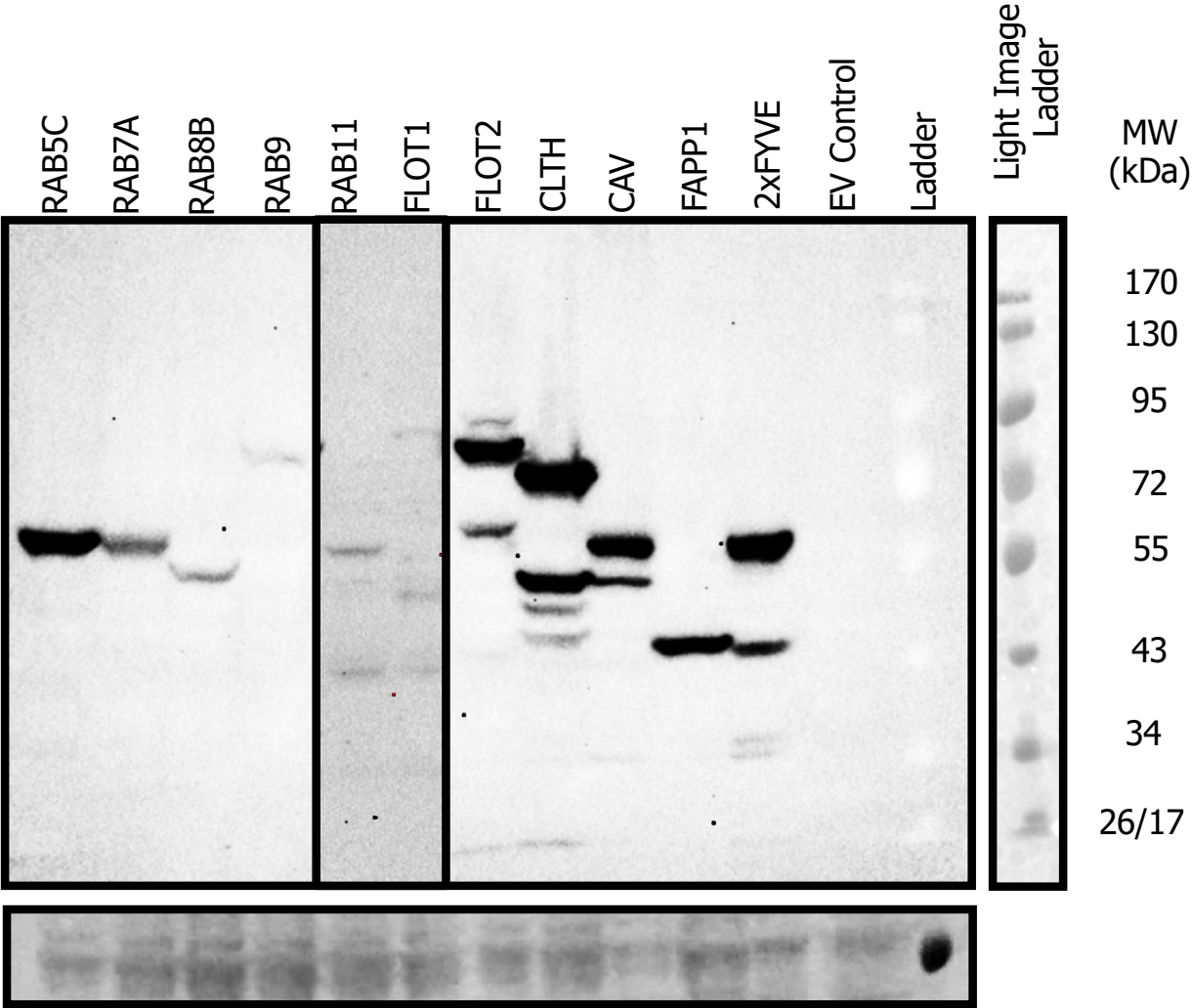

Supplement: FIG S6 [file mSphere.00663-18-sf006.pdf]
