# Supplementary material for: Endocytic Markers Associated with the Internalization and Processing of Aspergillus fumigatus Conidia by BEAS-2B Cells
Source: mSphere. 2019 Feb 6;4(1):e00663-18. doi: 10.1128/mSphere.00663-18 (PMC6365614; doi:10.1128/mSphere.00663-18)
Supplement: TEXT S1 [file mSphere.00663-18-s0001.docx]

>Clathrin

ACAAGTTTGTACAAAAAAGCAGGCTCCGCTGAACTGGACCCTTTCGGCGCACCCGCTGGGGCACCCGGAGGACCCGCACTGGGGAACGGAGTCGCTGGGGCTGGGGAAGAAGACCCCGCCGCTGCATTCCTGGCTCAGCAGGAGTCCGAAATCGCAGGAATTGAGAACGACGAAGCTTTTGCAATCCTGGATGGAGGAGCTCCAGGACCACAGCCTCACGGAGAGCCCCCTGGAGGCCCTGACGCCGTGGATGGGGTCATGAACGGAGAGTACTATCAGGAATCCAATGGCCCTACCGACTCATACGCCGCTATCAGCCAGGTGGATAGGCTGCAGTCAGAGCCAGAAAGCATTAGGAAATGGCGCGAGGAACAGATGGAGAGACTGGAAGCCCTGGACGCTAATTCTAGGAAACAGGAGGCAGAATGGAAGGAGAAAGCCATCAAGGAACTGGAGGAATGGTATGCCCGCCAGGATGAGCAGCTGCAGAAGACCAAAGCCAACAATCGAGCAGCCGAGGAAGCTTTCGTGAACGACATTGATGAGAGCTCCCCAGGGACAGAGTGGGAACGAGTCGCACGGCTGTGCGACTTTAATCCCAAGTCTAGTAAACAGGCCAAGGATGTGTCCCGGATGAGATCTGTCCTGATTAGTCTGAAGCAGGCCCCCCTGGTGCATACCCAGCTTTCTTGTACAAAGTGGT

>Clathrin-mCherry

MGTSLYKKAGSAELDPFGAPAGAPGGPALGNGVAGAGEEDPAAAFLAQQESEIAGIENDEAFAILDGGAPGPQPHGEPPGGPDAVDGVMNGEYYQESNGPTDSYAAISQVDRLQSEPESIRKWREEQMERLEALDANSRKQEAEWKEKAIKELEEWYARQDEQLQKTKANNRAAEEAFVNDIDESSPGTEWERVARLCDFNPKSSKQAKDVSRMRSVLISLKQAPLVHTQLSCTKWSLEVSKGEEDNMAIIKEFMRFKVHMEGSVNGHEFEIEGEGEGRPYEGTQTAKLKVTKGGPLPFAWDILSPQFMYGSKAYVKHPADIPDYLKLSFPEGFKWERVMNFEDGGVVTVTQDSSLQDGEFIYKVKLRGTNFPSDGPVMQKKTMGWEASSERMYPEDGALKGEIKQRLKLKDGGHYDAEVKTTYKAKKPVQLPGAYNVNIKLDITSHNEDYTIVEQYERAEGRHSTGGMDELYLEHHHHHH-

Theoretical pI/Mw: 5.05 / 53442.41

>Caveolin

ACAAGTTTGTACAAAAAAGCAGGCTCCAGCGGGGGAAAATACGTGGACTCCGAAGGACATCTGTACACCGTGCCCATCCGAGAACAGGGGAACATCTATAAACCCAATAATAAGGCCATGGCTGACGAGCTGAGCGAAAAGCAGGTGTACGATGCCCACACAAAAGAGATCGACCTGGTCAACAGGGATCCCAAGCATCTGAATGACGATGTGGTCAAAATCGACTTCGAAGATGTGATTGCCGAGCCTGAAGGCACTCACTCTTTTGACGGGATTTGGAAGGCTAGTTTCACCACCTTCACCGTGACCAAATACTGGTTCTATCGGCTGCTGTCCGCACTGTTTGGAATCCCAATGGCCCTGATCTGGGGCATCTACTTCGCTATCCTGTCATTTCTGCACATTTGGGCAGTGGTCCCCTGCATTAAGAGCTTCCTGATCGAGATTCAGTGTATCAGCCGGGTGTACTCCATCTACGTGCATACCGTCTGCGATCCCCTGTTCGAGGCCGTGGGGAAGATCTTTTCTAACGTCAGAATTAATCTGCAGAAAGAAATCACCCAGCTTTCTTGTACAAAGTGGT

>Caveolin-mCherry

MGTSLYKKAGSSGGKYVDSEGHLYTVPIREQGNIYKPNNKAMADELSEKQVYDAHTKEIDLVNRDPKHLNDDVVKIDFEDVIAEPEGTHSFDGIWKASFTTFTVTKYWFYRLLSALFGIPMALIWGIYFAILSFLHIWAVVPCIKSFLIEIQCISRVYSIYVHTVCDPLFEAVGKIFSNVRINLQKEITQLSCTKWSLEVSKGEEDNMAIIKEFMRFKVHMEGSVNGHEFEIEGEGEGRPYEGTQTAKLKVTKGGPLPFAWDILSPQFMYGSKAYVKHPADIPDYLKLSFPEGFKWERVMNFEDGGVVTVTQDSSLQDGEFIYKVKLRGTNFPSDGPVMQKKTMGWEASSERMYPEDGALKGEIKQRLKLKDGGHYDAEVKTTYKAKKPVQLPGAYNVNIKLDITSHNEDYTIVEQYERAEGRHSTGGMDELYLEHHHHHH-

Theoretical pI/Mw: 6.66 / 49065.64

>2xFYVE

ACAAGTTTGTACAAAAAAGCAGGCTCCATGGAATTTGAAAGCGATGCGATGTTTGCGGCGGAAAGAGCACCAGATTGGGTCGATGCGGAAGAATGTCACAGATGTCGTGTCCAGTTTGGTGTTGTGACCAGAAAACATCACTGTAGGGCCTGCGGACAGATTTTCTGTGGTAAATGCTCTTCAAAGTATAGCACTATTCCAAAATTCGGCATCGAAAAAGAAGTTAGAGTCTGTGAACCTTGCTATGAACAACTGAATAAAAAGGCTCAGGGTCAAGGCTCCGAATCTGATGCAATGTTTGCTGCAGAACGCGCCCCGGATTGGGTTGACGCGGAAGAGTGTCATCGTTGCCGCGTGCAGTTCGGAGTCGTTACACGTAAACATCACTGTCGCGCATGCGGCCAAATCTTTTGTGGAAAATGCAGCAGTAAGTATTCAACCATCCCTAAGTTTGGTATTGAAAAAGAAGTTCGTGTGTGCGAGCCGTGTTACGAGCAACTGAATAAAAAGGCCGTGGACCTGCAACCGAAGACCCAGCTTTCTTGTACAAAGTGGT

>2xFYVE-mCherry

MGTSLYKKAGSMEFESDAMFAAERAPDWVDAEECHRCRVQFGVVTRKHHCRACGQIFCGKCSSKYSTIPKFGIEKEVRVCEPCYEQLNKKAQGQGSESDAMFAAERAPDWVDAEECHRCRVQFGVVTRKHHCRACGQIFCGKCSSKYSTIPKFGIEKEVRVCEPCYEQLNKKAVDLQPKTQLSCTKWSLEVSKGEEDNMAIIKEFMRFKVHMEGSVNGHEFEIEGEGEGRPYEGTQTAKLKVTKGGPLPFAWDILSPQFMYGSKAYVKHPADIPDYLKLSFPEGFKWERVMNFEDGGVVTVTQDSSLQDGEFIYKVKLRGTNFPSDGPVMQKKTMGWEASSERMYPEDGALKGEIKQRLKLKDGGHYDAEVKTTYKAKKPVQLPGAYNVNIKLDITSHNEDYTIVEQYERAEGRHSTGGMDELYLEHHHHHH-

Theoretical pI/Mw: 6.66 / 49065.64

>FAPP1

ACAAGTTTGTACAAAAAAGCAGGCTCCATGGAAGGTGTTCTGTATAAATGGACAAATTACCTGACCGGCTGGCAGCCACGTTGGTTTGTTCTGGATAACGGTATTCTGTCTTATTACGATTCACAAGATGATGTGTGTAAAGGTAGCAAAGGCAGTATCAAGATGGCTGTTTGCGAAATCAAGGTGCATTCTGCAGATAATACCCGTATGGAACTGATTATTCCTGGCGAACAGCATTTCTATATGAAAGCTGTTAACGCTGCAGAACGTCAACGCTGGCTGGTTGCTCTGGGTTCTTCAAAAGCGTGTCTGACCGATACTCGCACCCAGCTTTCTTGTACAAAGTGGT

>FAPP1-mCherry

MGTSLYKKAGSMEGVLYKWTNYLTGWQPRWFVLDNGILSYYDSQDDVCKGSKGSIKMAVCEIKVHSADNTRMELIIPGEQHFYMKAVNAAERQRWLVALGSSKACLTDTRTQLSCTKWSLEVSKGEEDNMAIIKEFMRFKVHMEGSVNGHEFEIEGEGEGRPYEGTQTAKLKVTKGGPLPFAWDILSPQFMYGSKAYVKHPADIPDYLKLSFPEGFKWERVMNFEDGGVVTVTQDSSLQDGEFIYKVKLRGTNFPSDGPVMQKKTMGWEASSERMYPEDGALKGEIKQRLKLKDGGHYDAEVKTTYKAKKPVQLPGAYNVNIKLDITSHNEDYTIVEQYERAEGRHSTGGMDELYLEHHHHHH-

Theoretical pI/Mw: 6.29 / 41247.70

>Flot2

ACAAGTTTGTACAAAAAAGCAGGCTCCATGACGTTGCAGCCCCGCTGCGAGGACGTAGAGACGGCCGAGGGGGTAGCTTTAACTGTGACGGGTGTCGCCCAGGTGAAGATCATGACGGAGAAGGAACTCCTGGCCGTGGCTTGTGAGCAGTTTCTGGGTAAGAATGTGCAGGACATCAAAAACGTCGTCCTGCAGACCCTGGAGGGACATCTGCGCTCCATCCTCGGGACCCTGACAGTGGAGCAGATTTATCAGGACCGGGACCAGTTTGCCAAGCTGGTGCGGGAGGTGGCAGCCCCTGATGTTGGCCGCATGGGCATTGAGATCCTCAGCTTCACCATCAAGGACGTGTATGACAAAGTGGACTATCTGAGCTCCCTGGGCAAGACGCAGACTGCCGTGGTGCAGAGAGATGCTGACATTGGCGTGGCCGAGGCTGAACGGGACGCAGGCATCCGGGAAGCTGAGTGCAAGAAGGAGATGCTGGATGTGAAGTTCATGGCAGACACCAAGATTGCTGACTCTAAGCGAGCCTTCGAGCTGCAAAAGTCAGCCTTCAGTGAGGAGGTTAACATCAAGACAGCTGAGGCCCAGTTGGCCTATGAGCTGCAGGGGGCCCGTGAACAGCAGAAGATCCGGCAGGAAGAGATTGAGATTGAGGTTGTGCAGCGCAAGAAACAGATTGCCGTGGAGGCACAGGAGATCCTGCGTACGGACAAGGAGCTCATCGCTACAGTGCGCCGGCCTGCCGAGGCCGAGGCCCACCGCATCCAGCAGATTGCCGAGGGTGAAAAGGTGAAGCAGGTCCTCTTGGCACAGGCAGAGGCTGAGAAGATCCGCAAAATCGGGGAGGCGGAAGCGGCAGTCATCGAGGCGATGGGCAAGGCAGAGGCTGAGCGGATGAAGCTCAAGGCAGAAGCCTACCAGAAATACGGGGATGCAGCCAAGATGGCCTTGGTGCTAGAGGCCCTGCCCCAGATTGCTGCCAAAATCGCTGCCCCACTTACCAAGGTCGATGAGATTGTGGTCCTCAGTGGAGACAACAGTAAGGTCACATCAGAAGTGAACCGACTGCTGGCCGAGCTGCCTGCCTCTGTGCATGCCCTCACAGGCGTGGACCTGTCTAAGATACCCCTGATCAAGAAGGCCACTGGTGTGCAGGTGACCCAGCTTTCTTGTACAAAGTGGT

>FLot2-mCherry

MGTSLYKKAGSMTLQPRCEDVETAEGVALTVTGVAQVKIMTEKELLAVACEQFLGKNVQDIKNVVLQTLEGHLRSILGTLTVEQIYQDRDQFAKLVREVAAPDVGRMGIEILSFTIKDVYDKVDYLSSLGKTQTAVVQRDADIGVAEAERDAGIREAECKKEMLDVKFMADTKIADSKRAFELQKSAFSEEVNIKTAEAQLAYELQGAREQQKIRQEEIEIEVVQRKKQIAVEAQEILRTDKELIATVRRPAEAEAHRIQQIAEGEKVKQVLLAQAEAEKIRKIGEAEAAVIEAMGKAEAERMKLKAEAYQKYGDAAKMALVLEALPQIAAKIAAPLTKVDEIVVLSGDNSKVTSEVNRLLAELPASVHALTGVDLSKIPLIKKATGVQVTQLSCTKWSLEVSKGEEDNMAIIKEFMRFKVHMEGSVNGHEFEIEGEGEGRPYEGTQTAKLKVTKGGPLPFAWDILSPQFMYGSKAYVKHPADIPDYLKLSFPEGFKWERVMNFEDGGVVTVTQDSSLQDGEFIYKVKLRGTNFPSDGPVMQKKTMGWEASSERMYPEDGALKGEIKQRLKLKDGGHYDAEVKTTYKAKKPVQLPGAYNVNIKLDITSHNEDYTIVEQYERAEGRHSTGGMDELYLEHHHHHH-

Theoretical pI/Mw: 5.60 / 71596.79

>Flot1

ACAAGTTTGTACAAAAAAGCAGGCTCCATGTTTTTCACTTGTGGCCCAAATGAGGCCATGGTGGTCTCCGGGTTCTGCCGAAGCCCCCCAGTCATGGTGGCTGGAGGGCGTGTCTTTGTCCTGCCCTGCATCCAACAGATCCAGAGGATCTCTCTCAACACACTGACCCTCAATGTCAAGAGTGAAAAGGTTTACACTCGCCATGGGGTCCCCATCTCAGTCACTGGCATTGCCCAGGTAAAAATCCAGGGGCAGAACAAGGAGATGTTGGCGGCCGCCTGTCAGATGTTCCTGGGGAAGACGGAGGCTGAGATTGCCCACATTGCCCTGGAGACGTTAGAGGGCCACCAGAGGGCCATCATGGCCCACATGACTGTGGAGGAGATCTATAAGGACAGGCAGAAATTCTCAGAACAGGTTTTCAAAGTGGCCTCCTCAGACCTGGTCAACATGGGCATCAGTGTGGTTAGCTACACTCTGAAGGACATTCACGATGACCAGGACTATTTGCACTCTTTGGGGAAGGCTCGAACAGCTCAAGTCCAAAAAGATGCACGGATTGGAGAAGCAGAGGCCAAGAGAGATGCTGGGATCCGGGAAGCTAAAGCCAAGCAGGAAAAGGTGTCTGCTCAGTACCTGAGTGAGATCGAGATGGCCAAGGCACAGAGAGATTACGAACTGAAGAAGGCCGCCTATGACATCGAGGTCAACACCCGCCGAGCACAGGCTGACCTGGCCTATCAGCTTCAGGTGGCCAAGACTAAGCAGCAGATTGAGGAGCAGCGGGTGCAGGTGCAGGTGGTGGAGCGGGCCCAGCAGGTGGCAGTGCAGGAGCAGGAGATCGCCCGGCGGGAGAAGGAGCTGGAGGCCCGGGTGCGGAAGCCAGCGGAAGCGGAGCGCTACAAGCTGGAGCGCCTAGCCGAGGCAGAGAAGTCCCAACTAATTATGCAGGCGGAGGCAGAAGCCGCGTCTGTGCGGATGCGTGGGGAAGCTGAGGCCTTTGCCATAGGGGCCCGAGCCCGAGCCGAGGCTGAGCAGATGGCCAAGAAGGCAGAAGCCTTCCAGCTGTACCAAGAGGCTGCTCAGCTGGACATGCTGCTAGAGAAGCTGCCCCAGGTGGCAGAGGAGATCAGTGGTCCCTTGACTTCAGCCAATAAGATCACACTGGTGTCCAGCGGCAGTGGGACCATGGGGGCAGCCAAAGTGACTGGGGAAGTACTGGACATTCTAACTCGCCTGCCAGAGAGTGTGGAAAGACTCACAGGCGTGAGCATCTCCCAGGTGAATCACAAGCCTTTGAGAACAGCCACCCAGCTTTCTTGTACAAAGTGGT

>Flot1-mCherry

MGTSLYKKAGSMFFTCGPNEAMVVSGFCRSPPVMVAGGRVFVLPCIQQIQRISLNTLTLNVKSEKVYTRHGVPISVTGIAQVKIQGQNKEMLAAACQMFLGKTEAEIAHIALETLEGHQRAIMAHMTVEEIYKDRQKFSEQVFKVASSDLVNMGISVVSYTLKDIHDDQDYLHSLGKARTAQVQKDARIGEAEAKRDAGIREAKAKQEKVSAQYLSEIEMAKAQRDYELKKAAYDIEVNTRRAQADLAYQLQVAKTKQQIEEQRVQVQVVERAQQVAVQEQEIARREKELEARVRKPAEAERYKLERLAEAEKSQLIMQAEAEAASVRMRGEAEAFAIGARARAEAEQMAKKAEAFQLYQEAAQLDMLLEKLPQVAEEISGPLTSANKITLVSSGSGTMGAAKVTGEVLDILTRLPESVERLTGVSISQVNHKPLRTATQLSCTKWSLEVSKGEEDNMAIIKEFMRFKVHMEGSVNGHEFEIEGEGEGRPYEGTQTAKLKVTKGGPLPFAWDILSPQFMYGSKAYVKHPADIPDYLKLSFPEGFKWERVMNFEDGGVVTVTQDSSLQDGEFIYKVKLRGTNFPSDGPVMQKKTMGWEASSERMYPEDGALKGEIKQRLKLKDGGHYDAEVKTTYKAKKPVQLPGAYNVNIKLDITSHNEDYTIVEQYERAEGRHSTGGMDELYLEHHHHHH-

Theoretical pI/Mw: 6.39 / 77266.99

>Rab5c

ACAAGTTTGTACAAAAAAGCAGGCTCCATGGCGGGTCGGGGAGGCGCAGCACGACCCAATGGACCAGCTGCTGGGAACAAGATCTGTCAATTTAAGCTGGTTCTGCTGGGGGAGTCTGCGGTAGGCAAATCCAGCCTCGTCCTCCGCTTTGTCAAGGGACAGTTTCACGAGTACCAGGAGAGCACAATTGGAGCGGCCTTCCTCACACAGACTGTCTGCCTGGATGACACAACAGTCAAGTTTGAGATCTGGGACACAGCTGGACAGGAGCGGTATCACAGCCTGGCCCCCATGTACTATCGGGGGGCCCAGGCTGCCATCGTGGTCTATGACATCACCAACACAGATACATTTGCACGGGCCAAGAACTGGGTGAAGGAGCTACAGAGGCAGGCCAGCCCCAACATCGTCATTGCACTCGCGGGTAACAAGGCAGACCTGGCCAGCAAGAGAGCCGTGGAATTCCAGGAAGCACAAGCCTATGCAGACGACAACAGTTTGCTGTTCATGGAGACATCAGCAAAGACTGCAATGAACGTGAACGAAATCTTCATGGCAATAGCTAAGAAGCTTCCCAAGAACGAGCCCCAGAATGCAACTGGTGCTCCAGGCACCCAGCTTTCTTGTACAAAGTGGT

>Rab5c-mCherry

MGTSLYKKAGSMAGRGGAARPNGPAAGNKICQFKLVLLGESAVGKSSLVLRFVKGQFHEYQESTIGAAFLTQTVCLDDTTVKFEIWDTAGQERYHSLAPMYYRGAQAAIVVYDITNTDTFARAKNWVKELQRQASPNIVIALAGNKADLASKRAVEFQEAQAYADDNSLLFMETSAKTAMNVNEIFMAIAKKLPKNEPQNATGAPGRNRGVDLQENNPASRSQCCSNTQLSCTKWSLEVSKGEEDNMAIIKEFMRFKVHMEGSVNGHEFEIEGEGEGRPYEGTQTAKLKVTKGGPLPFAWDILSPQFMYGSKAYVKHPADIPDYLKLSFPEGFKWERVMNFEDGGVVTVTQDSSLQDGEFIYKVKLRGTNFPSDGPVMQKKTMGWEASSERMYPEDGALKGEIKQRLKLKDGGHYDAEVKTTYKAKKPVQLPGAYNVNIKLDITSHNEDYTIVEQYERAEGRHSTGGMDELYLEHHHHHH*

Theoretical pI/Mw: 6.56 / 53394.26

>Rab7a

ACAAGTTTGTACAAAAAAGCAGGCTCCATGACCTCTAGGAAGAAAGTGTTGCTGAAGGTTATCATCCTGGGAGATTCTGGAGTCGGGAAGACATCACTCATGAACCAGTATGTGAATAAGAAATTCAGCAATCAGTACAAAGCCACAATAGGAGCTGACTTTCTGACCAAGGAGGTGATGGTGGATGACAGGCTAGTCACAATGCAGATATGGGACACAGCAGGACAGGAACGGTTCCAGTCTCTCGGTGTGGCCTTCTACAGAGGTGCAGACTGCTGCGTTCTGGTATTTGATGTGACTGCCCCCAACACATTCAAAACCCTAGATAGCTGGAGAGATGAGTTTCTCATCCAGGCCAGTCCCCGAGATCCTGAAAACTTCCCATTTGTTGTGTTGGGAAACAAGATTGACCTCGAAAACAGACAAGTGGCCACAAAGCGGGCACAGGCCTGGTGCTACAGCAAAAACAACATTCCCTACTTTGAGACCAGTGCCAAGGAGGCCATCAACGTGGAGCAGGCGTTCCAGACGATTGCACGGAATGCACTTAAGCAGGAAACGGAGGTGGAGCTGTACAACGAATTTCCTGAACCTATCAAACTGGACAAGAATGACCGGGCCAAGGCCTCGGCAGAAAGCTGCAGTTGCACCCAGCTTTCTTGTACAAAGTGGT

>Rab7a-mCherry

MGTSLYKKAGSMTSRKKVLLKVIILGDSGVGKTSLMNQYVNKKFSNQYKATIGADFLTKEVMVDDRLVTMQIWDTAGQERFQSLGVAFYRGADCCVLVFDVTAPNTFKTLDSWRDEFLIQASPRDPENFPFVVLGNKIDLENRQVATKRAQAWCYSKNNIPYFETSAKEAINVEQAFQTIARNALKQETEVELYNEFPEPIKLDKNDRAKASAESCSCTQLSCTKWSLEVSKGEEDNMAIIKEFMRFKVHMEGSVNGHEFEIEGEGEGRPYEGTQTAKLKVTKGGPLPFAWDILSPQFMYGSKAYVKHPADIPDYLKLSFPEGFKWERVMNFEDGGVVTVTQDSSLQDGEFIYKVKLRGTNFPSDGPVMQKKTMGWEASSERMYPEDGALKGEIKQRLKLKDGGHYDAEVKTTYKAKKPVQLPGAYNVNIKLDITSHNEDYTIVEQYERAEGRHSTGGMDELYLEHHHHHH-

Theoretical pI/Mw: 6.12 / 53401.45

>Rab8b

ACAAGTTTGTACAAAAAAGCAGGCTCCATGGCGAAGACGTACGATTATCTCTTCAAGCTCCTGCTGATCGGCGACTCGGGGGTAGGCAAGACCTGCCTCCTGTTCCGCTTCTCAGAGGACGCCTTCAACACCACCTTCATCTCCACCATCGGAATTGATTTTAAAATTAGAACGATAGAACTAGATGGAAAGAAAATTAAGCTTCAGATATGGGACACAGCGGGTCAGGAAAGATTCCGAACAATCACGACAGCGTACTACAGAGGAGCCATGGGCATTATGCTGGTCTATGACATCACAAATGAAAAATCCTTTGACAATATTAAAAATTGGATCAGAAACATTGAAGAGCATGCCTCTTCCGATGTCGAAAGAATGATCCTGGGTAACAAATGTGATATGAATGACAAAAGACAAGTGTCAAAAGAAAGAGGGGAGAAGCTAGCAATTGACTATGGGATTAAATTCTTGGAGACAAGCGCAAAATCCAGTGCAAATGTAGAAGAGGCATTTTTTACACTTGCACGAGATATAATGACAAAACTCAACAGAAAAATGAATGACAGCAATTCAGCAGGAGCAGGTGGACCAGTGAAAATAACAGAAAACCGATCAAAGAAGACCAGTTTCTTTCGTTGCTCGCTACTTACCCAGCTTTCTTGTACAAAGTGGT

>Rab8b-mCherry

MGTSLYKKAGSMAKTYDYLFKLLLIGDSGVGKTCLLFRFSEDAFNTTFISTIGIDFKIRTIELDGKKIKLQIWDTAGQERFRTITTAYYRGAMGIMLVYDITNEKSFDNIKNWIRNIEEHASSDVERMILGNKCDMNDKRQVSKERGEKLAIDYGIKFLETSAKSSANVEEAFFTLARDIMTKLNRKMNDSNSAGAGGPVKITENRSKKTSFFRCSLLTQLSCTKWSLEVSKGEEDNMAIIKEFMRFKVHMEGSVNGHEFEIEGEGEGRPYEGTQTAKLKVTKGGPLPFAWDILSPQFMYGSKAYVKHPADIPDYLKLSFPEGFKWERVMNFEDGGVVTVTQDSSLQDGEFIYKVKLRGTNFPSDGPVMQKKTMGWEASSERMYPEDGALKGEIKQRLKLKDGGHYDAEVKTTYKAKKPVQLPGAYNVNIKLDITSHNEDYTIVEQYERAEGRHSTGGMDELYLEHHHHHH-

Theoretical pI/Mw: 7.22 / 53495.80

>Rab9

ACAAGTTTGTACAAAAAAGCAGGCTCCATGAAGCAACTGCCAGTCTTGGAACCTGGAGACAAGCCCAGGAAAGCAACATGGTACACCTTGACTGTCCCTGGAGACAGCCCCTGTGCTCGAGTTGGCCACAGCTGTTCATATTTACCCCCAGTTGGTAATGCCAAGAGAGGGAAGGTCTTCATTGTTGGGGGAGCAAATCCAAACAGAAGCTTCTCAGACGTGCACACCATGGATCTGGGAAAACACCAGTGGGACTTAGATACCTGCAAGGGCCTCTTGCCCCGGTATGAACATGCTAGCTTCATTCCCTCCTGCACACCTGACCGTATCTGGGTATTTGGAGGTGCCAACCAATCAGGAAATCGAAATTGTCTACAAGTCCTGAATCCTGAAACCAGGACGTGGACCACGCCAGAAGTGACCAGCCCCCCACCATCCCCAAGAACATTCCACACATCATCGGCAGCCATTGGAAACCAGCTATATGTCTTTGGGGGCGGAGAGAGAGGTGCCCAGCCCGTGCAGGACACGAAGCTGCATGTGTTTGACGCAAACACTCTGACCTGGTCACAGCCAGAGACACTTGGAAATCCTCCATCTCCCCGGCATGGTCATGTGATGGTGGCAGCAGGGACAAAGCTCTTCATCCACGGAGGCTTGGCGGGGGACAGATTCTATGATGACCTCCACTGCATTGATATAAGTGACATGAAATGGCAGAAGCTAAATCCCACTGGGGCTGCTCCAGCAGGCTGTGCTGCCCACTCAGCTGTGGCCATGGGAAAACATGTGTACATCTTTGGTGGAATGACTCCTGCAGGAGCACTGGACACAATGTACCAGTATCACACAGAAGAGCAGCATTGGACCTTGCTTAAATTTGATACTCTTCTACCCCCTGGACGATTGGACCATTCCATGTGTATCATTCCATGGCCAGTGACGTGTGCTTCTGAGAAAGAAGATTCCAACTCTCTCACTCTGAACCATGAAGCTGAGAAAGAGGATTCAGCTGACAAAGTAATGAGCCACAGTGGTGACTCACATGAGGAAAGCCAGACTGCTACACTGCTCTGTTTGGTGTTTGGTGGGATGAATACAGAAGGGGAAATCTATGACGATTGTATTGTGACTGTAGTGGACACCCAGCTTTCTTGTACAAAGTGGT

>Rab9-mCherry

MGTSLYKKAGSMKQLPVLEPGDKPRKATWYTLTVPGDSPCARVGHSCSYLPPVGNAKRGKVFIVGGANPNRSFSDVHTMDLGKHQWDLDTCKGLLPRYEHASFIPSCTPDRIWVFGGANQSGNRNCLQVLNPETRTWTTPEVTSPPPSPRTFHTSSAAIGNQLYVFGGGERGAQPVQDTKLHVFDANTLTWSQPETLGNPPSPRHGHVMVAAGTKLFIHGGLAGDRFYDDLHCIDISDMKWQKLNPTGAAPAGCAAHSAVAMGKHVYIFGGMTPAGALDTMYQYHTEEQHWTLLKFDTLLPPGRLDHSMCIIPWPVTCASEKEDSNSLTLNHEAEKEDSADKVMSHSGDSHEESQTATLLCLVFGGMNTEGEIYDDCIVTVVDTQLSCTKWSLEVSKGEEDNMAIIKEFMRFKVHMEGSVNGHEFEIEGEGEGRPYEGTQTAKLKVTKGGPLPFAWDILSPQFMYGSKAYVKHPADIPDYLKLSFPEGFKWERVMNFEDGGVVTVTQDSSLQDGEFIYKVKLRGTNFPSDGPVMQKKTMGWEASSERMYPEDGALKGEIKQRLKLKDGGHYDAEVKTTYKAKKPVQLPGAYNVNIKLDITSHNEDYTIVEQYERAEGRHSTGGMDELYLEHHHHHH-

Theoretical pI/Mw: 5.91 / 70476.35

>Rab11

ACAAGTTTGTACAAAAAAGCAGGCTCCATGGGCACCCGCGACGACGAGTACGACTACCTCTTTAAAGTTGTCCTTATTGGAGATTCTGGTGTTGGAAAGAGTAATCTCCTGTCTCGATTTACTCGAAATGAGTTTAATCTGGAAAGCAAGAGCACCATTGGAGTAGAGTTTGCAACAAGAAGCATCCAGGTTGATGGAAAAACAATAAAGGCACAGATATGGGACACAGCAGGGCAAGAGCGATATCGAGCTATAACATCAGCATATTATCGTGGAGCTGTAGGTGCCTTATTGGTTTATGACATTGCTAAACATCTCACATATGAAAATGTAGAGCGATGGCTGAAAGAACTGAGAGATCATGCTGATAGTAACATTGTTATCATGCTTGTGGGCAATAAGAGTGATCTACGTCATCTCAGGGCAGTTCCTACAGATGAAGCAAGAGCTTTTGCAGAAAAGAATGAAGCAAATGTCAGACAGACGCGAAAAACCCAGCTTTCTTGTACAAAGTGGT,

>Rab11-mCherry

MGTSLYKKAGSMGTRDDEYDYLFKVVLIGDSGVGKSNLLSRFTRNEFNLESKSTIGVEFATRSIQVDGKTIKAQIWDTAGQERYRAITSAYYRGAVGALLVYDIAKHLTYENVERWLKELRDHADSNIVIMLVGNKSDLRHLRAVPTDEARAFAEKNEANVRQTRKTQLSCTKWSLEVSKGEEDNMAIIKEFMRFKVHMEGSVNGHEFEIEGEGEGRPYEGTQTAKLKVTKGGPLPFAWDILSPQFMYGSKAYVKHPADIPDYLKLSFPEGFKWERVMNFEDGGVVTVTQDSSLQDGEFIYKVKLRGTNFPSDGPVMQKKTMGWEASSERMYPEDGALKGEIKQRLKLKDGGHYDAEVKTTYKAKKPVQLPGAYNVNIKLDITSHNEDYTIVEQYERAEGRHSTGGMDELYLEHHHHHH-

Theoretical pI/Mw: 6.45 / 47570.63
